# Acupuncture as an Add-On Treatment for Functional Dyspepsia: A Systematic Review and Meta-Analysis

**DOI:** 10.3389/fmed.2021.682783

**Published:** 2021-07-26

**Authors:** Chan-Young Kwon, Seok-Jae Ko, Boram Lee, Jae Myung Cha, Jin Young Yoon, Jae-Woo Park

**Affiliations:** ^1^Department of Oriental Neuropsychiatry, Dong-Eui University College of Korean Medicine, Busan, South Korea; ^2^Department of Gastroenterology, College of Korean Medicine, Kyung Hee University, Seoul, South Krea; ^3^Clinical Medicine Division, Korea Institute of Oriental Medicine, Daejeon, South Korea; ^4^Department of Internal Medicine, Kyung Hee University School of Medicine, Seoul, South Korea

**Keywords:** acupuncture, functional dyspepsia, dyspepsia, systematic review, meta-analysis

## Abstract

**Background:** We aimed to critically evaluate the effectiveness and safety of acupuncture as an add-on therapy to conventional Western medication (WM) and assess the quality of evidence (QoE) of these findings.

**Methods:** A total of 12 English, Korean, and Chinese databases were searched on December 18, 2020. Randomized controlled trials (RCTs) assessing the effectiveness of acupuncture as an add-on therapy to conventional WM for functional dyspepsia (FD) were included. The primary outcome was the symptom score of FD. The risk of bias of the included studies and QoE were evaluated using the Cochrane Collaboration's risk of bias tool and Grading of Recommendations, Assessment, Development, and Evaluation method, respectively.

**Results:** A total of 22 RCTs were included. The total and individual FD symptom scores were significantly improved in the acupuncture combined with WM groups compared with the WM alone groups, except for in one study. The Nepean dyspepsia index score and total effective rate mostly improved significantly in the acupuncture group, regardless of the WM used and acupuncture type. FD-related biomarkers, such as ghrelin and gastrin levels, showed mixed results. The acupuncture group showed a significantly lower recurrence rate after 3–6 months of follow-up than the WM alone group. There were no differences in the incidence of adverse events between the two groups. The included studies generally had low methodological quality. The QoE for the main findings was generally very low to moderate.

**Conclusion:** Limited evidence suggests that acupuncture has the potential to improve FD treatment in combination with conventional WM. Furthermore, the methodological quality of the included studies and QoE of the main findings were generally low. Therefore, RCTs with a rigorous methodology, including sham acupuncture and multiethnic subjects, should be performed.

**Systematic Review Registration:** OSF registries [https://osf.io/mxren], PROSPERO [CRD42021226608].

## Introduction

Functional dyspepsia (FD) is a common functional gastrointestinal disorder. Its main symptoms include postprandial fullness, early satiation, epigastric pain, and epigastric burning, which are not fully explained by routine clinical evaluation (1). Generally, FD can be classified into postprandial distress syndrome (PDS) and epigastric pain syndrome (EPS) (1). Although the underlying pathology of FD is not fully understood, it is considered to be multifactorial. Moreover, upper gastrointestinal inflammation, gastric and duodenal disturbances, *Helicobacter pylori* infection, increased duodenal eosinophils, and psychological distress have been reported to be involved (2, 3). The prevalence of FD is reported at various levels around the world, ranging from 5 to 40%. Furthermore, based on the Rome III criteria, the prevalence rate has been reported as 9.8–20.2% in Western countries and 5.3–12.8% in Eastern countries (4). The main symptoms of FD are digestive and abdominal discomfort. While FD is not a life-threatening disease, it seriously impairs the quality of life (QoL) of patients and can be an economic and social burden (5, 6). Thus, FD poses serious public health problems at both individual and societal levels.

Conventional approaches to FD include proton pump inhibitors, *Helicobacter pylori* eradication treatment, antidepressants, and psychotherapy (3). However, interest in complementary and alternative medicine (CAM) approaches, such as herbal medicine or acupuncture, is increasing (7). For example, the Japanese Society of Gastroenterology's evidence-based clinical practice guidelines for FD published in 2015 recommend using herbal medicine along with anxiolytics and antidepressants as a second-line treatment (8). In addition, some researchers have suggested that acupuncture could be considered when constructing a comprehensive management strategy for FD, particularly for the management of EPS (9). The growing interest in CAM approaches for FD maybe because they are characterized as “holistic” approaches (7). In addition, the CAM approach is expected to play a role in complementing the limitations of conventional medicine in FD treatment (7). Therefore, if conventional medicine and CAM approaches are appropriately integrated, better treatment may be able to be provided to patients with FD. However, as many studies have pointed out, this process requires a careful, evidence-based approach (7).

Although several systematic reviews have already reported the effectiveness and safety of acupuncture (a typical non-pharmacological CAM treatment) for FD (10–13) a rigorous evaluation of the strength of evidence using the Grading of Recommendations, Assessment, Development, and Evaluation (GRADE) has not been performed. This approach is essential for promoting the development of integrative medicine for FD in terms of evidence-based medicine (EBM) (8). Therefore, we aimed to comprehensively review randomized controlled trials (RCTs) of acupuncture as an add-on treatment to conventional Western medication (WM) for FD, critically evaluate the effectiveness and safety, and assess the quality of evidence (QoE). Through this, we expect to promote the development of integrative medicine for FD in terms of EBM and provide clinical evidence that is helpful in decision-making for clinicians, patients, and policymakers.

## Materials and Methods

### Protocol and Registration

The protocol of this study was published as a research paper (14) and we conducted this review accordingly. We registered our study with PROSPERO (registration number: CRD42021226608) and OSF registries (URL: https://osf.io/mxren). This study was reported according to the Preferred Reporting Items for Systematic Reviews and Meta-Analyses (PRISMA) 2010 checklist (Supplementary File 1) (15).

### Eligibility Criteria

Only RCTs evaluating the effectiveness and safety of acupuncture as an adjunctive therapy to conventional WM for FD were included without limitation of the publication status (not only studies published in journals but also gray literature such as theses and conference proceedings) or language. In the study design, we included only parallel-group studies. Only those diagnosed with FD based on standardized diagnostic criteria, such as Rome criteria or clinical symptoms, were included regardless of age, sex, or ethnicity. Studies involving patients with organic causes of dyspepsia were excluded. As treatment interventions, we included all types of acupuncture (manual acupuncture, electroacupuncture, auriculotherapy, and acupressure) as add-on therapies to conventional WM for FD, such as acid suppressants, prokinetics, *Helicobacter pylori* eradication, fundic relaxants, or antidepressants. For the control interventions, we included only conventional WM for FD.

The primary outcome of our study was the symptom score of FD, measured using such as the Nepean dyspepsia index (NDI) (16), gastrointestinal symptom rating scale (17), dyspepsia symptom severity index (18), and visual analog scale. The secondary outcome measures were (a) total effective rate (TER); (b) QoL measured by factors such as the FD-QoL (19) and the 36-item Short-Form Health Survey (SF-36) (20); (c) level of gut peptide hormones such as motilin, ghrelin, and gastrin; (d) incidence of adverse events during the treatment period; and (e) recurrence rate. Among them, TER is a non-validated outcome measure that is processed secondarily according to evaluation criteria such as the improvement rates of other quantified outcomes or clinical symptom improvement. Regarding the outcome, participants are generally classified as “cured,” “markedly improved,” “improved,” or “non-responder” after treatment. The following formula is generally used to calculate TER: N1 + N2 + N3/N, where N1, N2, and N3 are the number of cured, markedly improved, and improved patients, respectively, and N is the total sample size.

### Information Sources and Search Strategy

The following 12 English, Korean, and Chinese electronic databases were searched from their inception to December 18, 2020, Medline (via PubMed), EMBASE (via Elsevier), the Cochrane Central Register of Controlled Trials, Allied and Complementary Medicine Database (via EBSCO), Cumulative Index to Nursing and Allied Health Literature (via EBSCO), Oriental Medicine Advanced Searching Integrated System, Korean studies Information Service System, Research Information Service System, Korean Medical Database, Korea Citation Index, China National Knowledge Infrastructure, and Wanfang data. We searched the reference lists of the included studies and trial registries, such as clinicaltrials.gov, to include all possible relevant literature. In addition, we set the search strategy as comprehensively as possible through consultation with FD and systematic review experts. The detailed search strategies for each database are described in Supplementary File 2.

### Study Selection and Data Extraction

Using EndNote X8 (Clarivate Analytics, Philadelphia, USA), we imported documents retrieved from each database and other sources. After removing any duplicates, we examined the eligibility of the searched articles by reviewing the titles and abstracts for the first inclusion. Subsequently, the full text of each article was reviewed for final inclusion.

The following information was extracted from the included studies using a standardized, pre-defined, pilot-tested Excel form: basic research information (the first author's name, year of publication, country, or study setting), sample size, details of participants, treatment and control intervention, duration of intervention, outcome measures, adverse events, and information for the assessment of the risk of bias. In addition, we used the Standards for Reporting Interventions in Clinical Trials of Acupuncture checklist to extract detail on the acupuncture treatment methods used in each study. We contacted the corresponding authors of the included studies via e-mail for further information if the data were insufficient or ambiguous. Study selection and data extraction were independently conducted by two researchers (CYK and BL). In case of disagreement between them, a consensus was reached through discussions with another researcher (SJK).

### Risk of Bias Assessment

We assessed the risk of bias of the included studies using the Cochrane Collaboration's risk of bias tool (21). We evaluated the random sequence generation, allocation concealment, blinding of participants and personnel, blinding of outcome assessors, completeness of outcome data, selective reporting, and other biases as “low risk,” “unclear risk,” or “high risk” (21). For the other bias, we evaluated it based on the statistical homogeneity of the baseline clinical characteristics such as mean age, sex, or disease severity between the treatment and control groups. The risk of bias assessment was independently conducted by two researchers (CYK and BL). In case of disagreement between them, a consensus was reached through discussions with another researcher (SJK).

### Data Analysis and Synthesis

Details of the participants, treatment and control interventions, and outcomes from all included studies are descriptively summarized. For studies that used the same type of treatment and control intervention, we quantitatively synthesized them with the same outcome measures using Review Manager software (version 5.4; Cochrane, London, UK). We presented continuous and binary outcomes using the mean difference (MD) and risk ratio (RR) with 95% confidence intervals (CIs). We assessed the heterogeneity between the studies included in the meta-analysis using the χ^2^ test and the *I*^2^ statistic. *I*^2^ values >50 and >75% were considered indicative of substantial and considerable heterogeneity, respectively. We pooled the results using a random-effects model if the included studies had significant heterogeneity (*I*^2^ value > 50%). In contrast, we used a fixed-effects model if the heterogeneity was not significant or if the number of studies included in the meta-analysis was less than five. This was done due to the estimate of the between-study variance being imprecise (22).

We conducted subgroup analyses according to the following to interpret the cause of heterogeneity: (a) type of conventional WM (acid suppressants, prokinetics, *Helicobacter pylori* eradication, fundic relaxants, or antidepressants), and (b) type of acupuncture (manual acupuncture, electroacupuncture, auriculotherapy, or acupressure). A sensitivity analysis was performed to identify the robustness of the results of the meta-analysis by excluding (a) studies with a high risk of bias and (b) outliers that were numerically distant from the rest of the data. If more than ten studies were included in each meta-analysis, we evaluated the evidence of publication bias using funnel plots.

### QoE Assessment

We used the GRADE method to evaluate the QoE for the main findings of the synthesized study results (23). The risk of bias, inconsistency, indirectness, imprecision of the results, and publication bias of the main findings were evaluated via https://gradepro.org/ as “very low,” “low,” “moderate,” or “high.” The QoE assessment was independently conducted by two researchers (CYK and BL). Any discrepancies were resolved by discussion with another researcher (SJK).

## Results

### Study Selection

**Figure 1:**
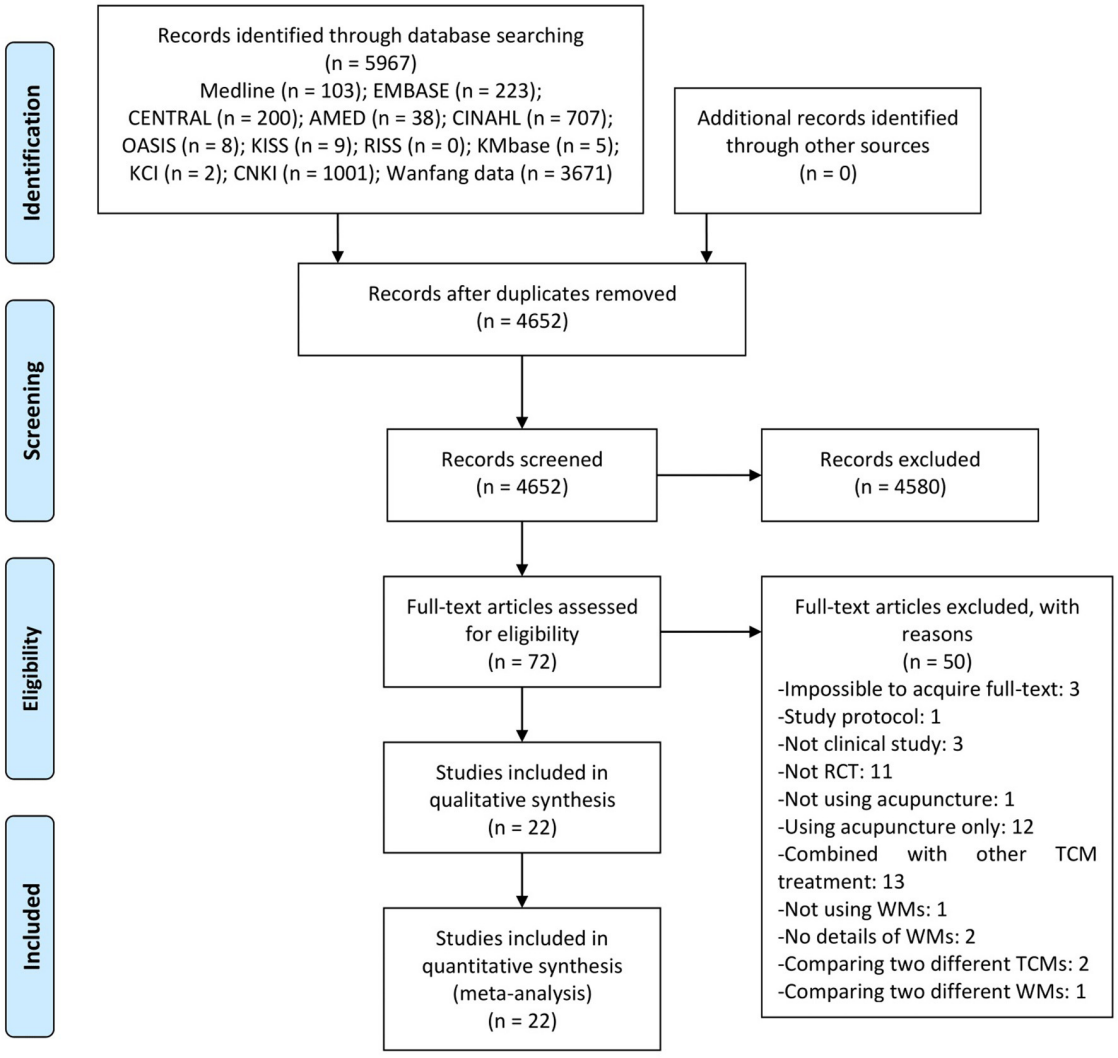
A PRISMA flow diagram of the literature screening and selection process. AMED, Allied and Complementary Medicine Database; CENTRAL, Cochrane Central Register of Controlled Trials; CINAHL, Cumulative Index to Nursing and Allied Health Literature; CNKI, China National Knowledge Infrastructure; KCI, Korea Citation Index; KISS, Koreanstudies Information Service System; KMbase, Korean Medical Database; OASIS, Oriental Medicine Advanced Searching Integrated System; RCT, randomized controlled trial; RISS, Research Information Service System; TCM, traditional Chinese medicine; WM, western medication.

A total of 5,967 studies were identified in our initial search. After any duplicates were removed, 4,652 studies remained. Then, using title and abstract screening, 72 potentially relevant articles were selected for inclusion. Following a full-text review, one, three, 11, one, 12, 13, one, two, two, one, and three studies were excluded due to being a study protocol, non-clinical studies, non-RCTs, did not use acupuncture, used acupuncture only, combined with other traditional Chinese medicine treatments, did not use WM, no details of WM compared to two different traditional Chinese medicine treatments, compared two different WM only, and unable to acquire the full text, respectively (Supplementary File 3). Finally, 22 RCTs were included in this review (Figure 1) (24–44).

### Study Characteristics

All included studies were conducted between 2008 and 2020. All studies were conducted by Chinese authors and were published in Chinese, except for one study (42) that was published in English in an international journal. Three studies were dissertations (24, 30, 31). Except for one article not mentioned (25), the study setting of all included studies was a hospital. Most studies used the Rome criteria to diagnose FD, including four studies (28, 29, 32, 34) using the Rome II criteria and 13 studies (24, 26, 27, 30–33, 35–39, 42) using the Rome III criteria. In eight studies, pattern identification was used and reflected in acupuncture treatment (24, 31–33, 37, 39, 40, 43). Han and Chen (44) performed a four-armed RCT and used two different WM protocols (protocol (A): mosapride and pantoprazole; protocol (B): mosapride, pantoprazole, and agomelatine) (44). Therefore, this study was classified into electroacupuncture combined with protocol (A) vs. protocol (A) and electroacupuncture combined with protocol (B) vs. protocol (B), which was named part one and part two of Han and Chen (44), respectively. Since these two parts do not overlap participants, we analyzed this study as two separate trials. (1) Eleven trials (27, 29–33, 35, 37, 38, 40, 41) compared acupuncture combined with prokinetics to prokinetics alone; (2) one trial (32) compared acupuncture combined with acid suppressants to acid suppressants alone; (3) seven trials (24–26, 28, 34, 43, 44) compared acupuncture combined with prokinetics and acid suppressants to prokinetics and acid suppressants; (4) two trials (36, 39) compared acupuncture combined with prokinetics and antidepressants to prokinetics and antidepressants; (5) one trial (44) compared acupuncture combined with prokinetics, acid suppressants, and antidepressants to prokinetics, acid suppressants, and antidepressants; and (6) one trial (42) compared acupuncture combined with gastrocaine (a potent local anesthetic for gastric pain) to gastrocaine alone. In addition, 17 trials used manual acupuncture (24–30, 32–36, 38, 39, 41, 43, 45), five used electroacupuncture (31, 40, 42, 44), and one used auricular acupuncture (37) (Table 1).

**Table 1:** General characteristics of the included studies.

**References**	**Sample size (included → analyzed)**	**Mean age (yr)**	**Disease period**	**Diagnosis criteria**	**Pattern identification**	**(A) Treatment intervention**	**(B) Control intervention**	**Outcomes**
Chen et al. (26)	128 (62:66)	42.0 ± 5.23	NR	Rome III	NR	(B) + MA	Rabeprazole + mosapride	1. TER (dyspepsia symptom)
Chen et al. (28)	112 (54:58)	38 (18–58)	8 mon−12 yr	Rome II	NR	(B) + MA	Lansoprazole + mosapride	1. TER (dyspepsia symptom)
Chen et al. (37)	90 (45:45)	(A) 67.1 ± 6.3 (62–74) (B) 66.8 ± 5.9 (60–75)	(A) 2.6 ± 1.1 yr (10 mon−4 yr) (B) 2.7 ± 0.8 yr (1–5 yr)	Rome III	Spleen-stomach weakness	(B) + EA	Mosapride	1. FD symptom score 2. Functional digestive disorder quality of life scale 3. TER (dyspepsia symptom)
Chen et al. (41)	100 (50:50)	(A) 50.82 ± 6.61 (B) 50.50 ± 6.58	(A) 2.10 ± 0.33 yr (B) 2.07 ± 0.31 yr	Consensus opinion on the diagnosis and treatment of functional dyspepsia with integrated TCM and Western medicine 2017	NR	(B) + MA	Mosapride	1. Serum motilin 2. Serum gastrin 3. FD symptom score 4. TER (dyspepsia symptom)
Chung et al. (42)	132 (66:66)	(A) 50.8 ± 11.2 (B) 47.6 ± 12.8	(A) 9.5 ± 9.3 yr (B) 9.3 ± 10.0 yr	Rome III	NR	(B) + EA	On-demand gastrocaine	1. Responder 2. Patient-reported global symptoms 3. Individual PDS symptoms 4. Other FD symptoms (epigastric pain, epigastric burning, and postprandial nausea) 5. Change of NDI score 6. Nutrient Drink Test 7. PHQ-9 8. GAD-7
Fan (33)	112 (56:56)	(A) 38.9 ± 7.9 (22–70) (B) 38.3 ± 7.8 (21–70)	(A) median 2.1 yr (0.7–6) (B) median 1.9 yr (0.5–5)	Rome III	Acupuncture treatment according to deficiency/excess pattern	(B) + MA	Mosapride	1. TER (dyspepsia symptom) 2. FD Symptom score 3. Electrogastrography
Gao (34)	100 (50:50)	(A) 38.24 ± 5.14 (19–56) (B) 38.19 ± 5.08 (20–57)	(A) 5.16 ± 1.24 yr (8 mon−12 yr) (B) 5.21 ± 1.33 yr (9 mon−13 yr)	Rome II	NR	(B) + MA	Lansoprazole + mosapride	1. TER (dyspepsia symptom) 2. SF-36
Han (44)	90 (45:45) → 79 (39:40)	NR	NR	NR	NR	(B) + EA	Mosapride + pantoprazole	1. PSQI 2. FD symptom score 3. SAS 4. SDS 5. Serum estrogen level 6. Plasma motilin level 7. TER (dyspepsia symptom)
Han (44)	90 (45:45) → 84 (43:41)	NR	NR	NR	NR	(B) + EA	Mosapride + pantoprazole + agomelatine	1. PSQI 2. FD symptom score 3. SAS 4. SDS 5. Serum estrogen level 6. Plasma motilin level 7. TER (dyspepsia symptom)
He (29)	260 (130:130)	(A) 46.2 ± 9.47 (B) 38.7 ± 9.86	(A) 2.7 ± 1.3 yr (B) 2.6 ± 1.4 yr	Rome II	NR	(B) + MA	Mosapride	1. TER (dyspepsia symptom) 2. FD symptom score 3. Recurrence rate (f/u for 3 mon after treatment)
Jiang (38)	90 (45:45)	(A) 50.15 ± 12.69 (B) 49.79 ± 13.75	(A) 16.53 ± 5.29 mon (B) 16.61 ± 4.75 mon	Rome III	NR	(B) + MA	Mosapride	1. PAGI-SYM 2. SF-36 3. Electrogastrography 4. TER (dyspepsia symptom)
Liu and Shu (27)	78 (40:38)	(A) 48.3 ± 4.8 (B) 46.0 ± 5.0	(A) 6.8 ± 1.1 mon (B) 7.0 ± 0.5 mon	Rome III	NR	(B) + MA	Clebopride	1. TER (dyspepsia symptom)
Mao (31)	80 (40:40)	(A) 45.28 ± 9.15 (B) 44.78 ± 10.20	(A) 19.18 ± 6.32 mon (B) 18.83 ± 7.48 mon	Rome III	Liver qi invading the stomach	(B) + EA	Mosapride	1. TCM symptom score 2. TER (TCM syndrome score) 3. FD symptom score 4. TER (dyspepsia symptom) 5. SAS 6. SDS 7. SF-36
Mei (40)	80 (40:40)	(A) 37.2 ± 5.3 (20–61) (B) 37.5 ± 4.8 (22–65)	(A) 6.3 ± 1.2 yr (1–18) (B) 6.8 ± 1.5 yr (1–15)	8th edition of the “Internal Medicine” textbook	Acupuncture treatment according to deficiency/excess pattern	(B) + EA	Domperidone	1. LDQ 2. NDI 3. Karnofsky performance scale
Wang (43)	42 (21:21)	(A) 34.52 ± 6.79 (B) 34.49 ± 6.72	(A) 4.52 ± 1.83 yr (B) 4.49 ± 1.79 yr	Consensus opinion on the diagnosis and treatment of functional dyspepsia with integrated TCM and western medicine 2010	Liver depression and spleen deficiency	(B) + MA	Domperidone + cimetidine	1. TER (dyspepsia symptom) 2. FD symptom score
Yan (35)	136 (68:68)	(A) 40.35 ± 6.41 (27–63) (B) 41.58 ± 7.34 (25–64)	(A) 6.68 ± 2.23 yr (6 mon−10 yr) (B) 6.87 ± 2.51 yr	Rome III	NR	(B) + MA	Mosapride	1. FD symptom score 2. TER (dyspepsia symptom) 3. Gastric emptying rate 4. Plasma motilin 5. Plasma ghrelin 6. Plasma 5-hydroxytryptamine
Yang et al. (36)	100 (50:50)	(A) 42.1 ± 13.9 (B) 40.8 ± 15.1	(A) 19.6 ± 10.3 mon (B) 23.6 ± 15.7 mon	Rome III PSQI ≥ 8	NR	(B) + MA	Itopride + deanxit	1. PSQI 2. Serum gastrin 3. TER (TCM syndrome score)
Yang and Huang (39)	100 (50:50)	(A) 43.5 ± 3.5 (21–65) (B) 44.2 ± 3.3 (22–65)	(A) 19.5 ± 5.5 mon (10 mon−3 yr) (B) 19.7 ± 5.3 mon (11 mon−3 yr)	Rome III PSQI ≥ 8	(A) liver qi depression (16), spleen-stomach qi deficiency (14), liver qi invading the stomach (13), dampness-heat stagnating in stomach (7) (B) liver qi depression (17), spleen-stomach qi deficiency (13), liver qi invading the stomach (12), dampness-heat stagnating in stomach (8)	(B) + MA	Itopride + flupentixol and melitroxine	1. PSQI 2. TER (sleep disorder) 3. SAS 4. SDS 5. SF-36 6. Serum motilin 7. Serum somatostatin8 Serum gastrin
Yu (24)	73 (37:36) → 70 (36:34)	(A) 39.7 ± 15.2 (18–66) (B) 41.4 ± 11.8 (18–65)	NR	Rome III depression and anxiety attacks (CCMD-3) 10 ≤ HAMD ≤ 30 7 ≤ HAMA ≤ 29	(A) liver qi stagnation (17), liver depression and spleen deficiency (8), spleen deficiency and phlegm-dampness (7), food accumulation and stagnation (2), cold-heat complex (2) (B) liver qi stagnation (16), liver depression and spleen deficiency (9), spleen deficiency and phlegm-dampness (7), food accumulation and stagnation (1), cold-heat complex (1)	(B) + MA	Domperidone + sucralfate	1. HAMD 2. HAMA 3. FD symptom score 4. TER (dyspepsia symptom)
Zhang (25)	61 (31:30)	(A) 47.3 ± 7.6 (B) 46.3 ± 8.1	NR	6th edition of the “Internal Medicine” textbook	NR	(B) + MA	Domperidone + omeprazole	1. TER (dyspepsia symptom)
Zhang (30)	72 (36:36)	(A) 30.63 ± 7.83 (B) 31.63 ± 7.84	(A) 17.27 ± 7.12 mon (B) 18.75 ± 6.9 mon	Rome III	NR	(B) + MA	Mosapride	1. FD symptom score 2. SF-36 3. TER (dyspepsia symptom)
Zhang (45)	58 (30:28)	(A) 33.60 ± 9.15 (21–60) (B) 31.78 ± 10.35 (18–58)	(A) 4.81 ± 2.67 yr (9 mon−12 yr) (B) 4.14 ± 1.80 yr (1–9 yr)	Rome III	liver qi invading the stomach	(B) + MA	Mosapride	1. FD symptom score 2. TER (dyspepsia symptom) 3. Recurrence rate (f/u for 3 mon after treatment)
Zhang (32)	76 (40:36)	NR	NR	Rome II	NR	(B) + MA	Rabeprazole	1. TER (dyspepsia symptom) 2. FD symptom score 3. Recurrence rate (f/u for 6 mon after treatment)

*EA, electro-acupuncture; FD, functional dyspepsia; GAD-7, general anxiety disorder-7; HAMA, Hamilton anxiety rating scale; HAMD, Hamilton depression rating scale; LDQ, Leeds indigestion symptom scores; MA, manual acupuncture; NDI, Nepean dyspepsia index; NR, not recorded; PAGI-SYM, patient assessment of upper gastrointestinal symptom severity; PDS, postprandial distress syndrome; PHQ-9, Patient health questionnaire-9; PSQI, Pittsburgh sleep quality index; SAS, self-rating anxiety scale; SDS, self-rating depression scale; SF-36, 36-item short-form health survey; TCM, traditional Chinese medicine; TER, total effective rate*.

Except for one trial (39) which set the main acupoints according to pattern identification, a total of 36 acupoints were used as the main acupoints in 22 trials. Among them, ST36 was used the most in 18 trials, followed by PC6 (16 trials), CV12 (13 trials), ST25 (six trials), BL20 (five trials), and LR3 (five trials). As a response to acupuncture, 13 trials acquired De qi, such as numbness, soreness, distention, and heaviness. In the study that used electroacupuncture, the frequency was varied from 2 to 100 Hz, and the intensity was performed to the extent that the participants could tolerate it. Continuous waves were used as the waveform in two trials (31, 42) and sparse and dense waves were used in one trial (40). The needle retention time was between 15 and 30 min, with 30 min the most commonly used (14 trials). The duration of treatment varied from 2 to 10 weeks, with 4 weeks the most common. The number of treatment sessions varied from 12 to 56, with 28 sessions being the most common (7 trials). In one trial (42) a full license from the Chinese Medicine Council of Hong Kong was presented as a qualification for acupuncture therapists (Table 2).

**Table 2:** Details of acupuncture method.

**References**	**Style of acupuncture**	**Number of needle (main acupoints)**	**Treatment points**	**Depth of insertion**	**Response sought**	**Needle stimulation**	**Needle retention time**	**Needle type**	**Number of treatment session**	**Frequency and duration**	**Qualification or experiences on acupuncture**
Chen et al. (26)	MA	Unclear	ST36, PC6	NR	NR	NR	30 min	NR	28	1 session/day for 4 wks	NR
Chen et al. (28)	MA	Unclear	ST36, PC6	NR	NR	NR	30 min	NR	28	1 session/day for 4 wks	NR
Chen et al. (37)	Auricular acupuncture	5	Spleen, stomach, large intestine, triple energizers, cardia	NR	NR	Press once every 4 h about 1 min, 3 times a day to the extent that the soreness or fever can be tolerated	2 days	0.20 × 1.5 mm	14	Every other day for 4 wks (alternating the left and right ears)	NR
Chen et al. (41)	MA	Unclear	CV6, CV4, CV11, ST36, CV12, PC6 -liver qi invading the stomach: LR3, LR14 -food damage to spleen: CV10, ST21 -spleen-stomach deficiency cold: SP6, LR13, BL20, BL21	NR	NR	NR	NR	NR	30	1 session/day for 1 mon	NR
Chung et al. (42)	EA	15	Bilateral ST34, ST36, ST40, ST42, CV12, PC6, BL20, BL21	Depends on the patient's body type	De qi	EA for all acupoints except BL20, BL21, ST42, and CV12. 2 Hz, continuous wave, acceptable to the patient (0.5–1.5 mA)	30 min, no retention for BL20 and BL21	0.20 × 25–40 mm	20	2 sessions/wk for 10 wks	Full licensure from the Chinese Medicine Council of Hong Kong
Fan (33)	MA	Unclear	BL21, BL20, CV11, ST36, ST23 -deficiency pattern: SP9, SP4 -excess pattern: ST44, LR3	NR	NR	Neutral supplementation and draining, needle manipulation every 15 min	30 min	Disposable acupuncture needle	24	1 session/day, 6 day/wk for 4 wks	NR
Gao (34)	MA	Unclear	ST36, PC6	NR	NR	NR	30 min	NR	28	1 session/day for 4 wks	NR
Han (44)	EA	9	Bilateral ST36, CV12, SP6, LR3, PC6	15–45 mm	De qi	G6805 EA therapy device, 2–100 Hz, patient's perception of mild muscle tremor	15 min	Huatuo needles, 0.38 × 50–80 mm, 28 gauge acupuncture	56	2 sessions/day for 4 wks	NR
Han (44)	MA	Unclear	ST36, ST44, LR3, PC6, BL20, BL21, BL18, BL15, CV12, et al.	NR	De qi	NR	15–30 min	NR	28	1 session/day for 4 wks	NR
He (29)	MA	15	Bilateral BL20, BL21, PC6, CV12, ST25, ST36, ST34, ST40 -deficiency pattern: Bilateral GB34, SP4 -excess pattern: Bilateral ST44, LR3	NR	De qi	NR	30 min	NR	20	5 sessions/wk for 4 wks	NR
Jiang (38)	MA	6	Bilateral ST36, PC6, ST25	NR	De qi	NR	20–30 min	NR	NR	4 wks	NR
Liu and Shu (27)	EA	7 or 9	CV12, bilateral PC6, ST36, CV6, LR3, LR14, BL18, ST37, ST25	NR	De qi	20 Hz, continuous wave, acceptable to the patient	20 min	0.25 × 40 mm	24	6 sessions/wk for 4 wks	NR
Mao (31)	EA	Unclear	ST36, PC6 -deficiency pattern: SP4, SP9 -excess pattern: LR3, ST44	NR	De qi	Sparse and dense wave, 2/100 Hz, 0.1–1.0 mA	30 min	Disposable acupuncture needle, 0.25 × 40 mm	30	1 session/day for 1 mon	NR
Mei (40)	Abdomen acupuncture	6	CV10, CV12, CV4, CV6, bilateral ST25	NR	Not de qi	NR	30 min	NR	12	6 sessions/wk for 2 wks	NR
Wang (43)	MA	8	CV12, CV10, bilateral ST36, ST21, PC6	NR	NR	Neutral supplementation and draining, needle manipulation for 1 min every 10 min	30 min	Disposable acupuncture needles, Huatuo needles, 0.30 × 50 mm	28	1 session/day for 4 wks	NR
Yan (35)	MA	Unclear	CV12, CV13, CV10, CV6, ST25, ST36, PC6, GV20, GV24, EX-HN3 -liver qi stagnation: LR3 -liver qi invading the stomach: SP4 -spleen-stomach weakness: CV4 -dampness-heat stasis and stagnation: GB34	NR	De qi	NR	30 min	NR	20	5 sessions/wk for 1 mon	NR
Yang et al. (36)	MA	Unclear	CV12, ST25, ST36, HT7, PC6, EX-HN1, SP6 -liver qi depression: CV17, LR13 -spleen-stomach qi deficiency: BL20, BL21 -liver qi invading the stomach: LR14, LR3 -dampness-heat stagnating in stomach: SP9, ST44	NR	Numbness, soreness, distention, and heaviness	60–90 times/min (lifting frequency), 90–180°(twisting angle), 60–90 times/min (twisting frequency)	30 min	Disposable stainless steel acupuncture needle	14	1 session/day for 2 wks	NR
Yang and Huang (39)	MA	12	Bilateral BL13, BL15, BL18, BL20, BL23, BL17	0.5–0.8 cun (寸)	De qi	Neutral supplementation and draining	30 min	Stainless steel acupuncture needles, Huatuo needles, 0.32 × 50–70 mm	24	3 sessions/wk for 8 wks	NR
Yu (24)	MA	Unclear	CV12, ST36 -liver qi stagnation, stomach qi failing to bear downward: BL18, LR14, ST34, PC6 -spleen deficiency: CV10, ST25	NR	NR	NR	NR	NR	NR	2 wks	NR
Zhang (25)	Abdomen acupuncture	7	CV4, CV12, CV10, bilareral ST25, SP15 -liver qi invading the stomach: Right CV4, ST23, up wind-dampness -spleen deficiency and qi stagnation, spleen-stomach deficiency cold: Left CV4, ST23, ST28, ST26 -vomiting: KI18, ST24 -insomnia: KI19, bilareral down wind-dampness	To the abdominal wall	Diffuse pain in the abdominal wall	NR	25 min	0.22 × 40 mm, 0.22 × 25 mm	15	1 session/day for 15 days	NR
Zhang (30)	MA	7	CV12, bilateral ST36, PC6, LR3	1–1.5 cun (寸)	De qi	Neutral supplementation and draining	30 min	Disposable sterile filiform needle	28	1 session/day for 4 wks	NR
Zhang (46)	MA	Unclear	PC6, ST36	NR	NR	NR	30 min	NR	28	7 sessions/wk for 4 wks	NR

*EA, electro-acupuncture; MA, manual acupuncture; NR, not recorded*.

### Risk of Bias Assessment

**Figure 2:**
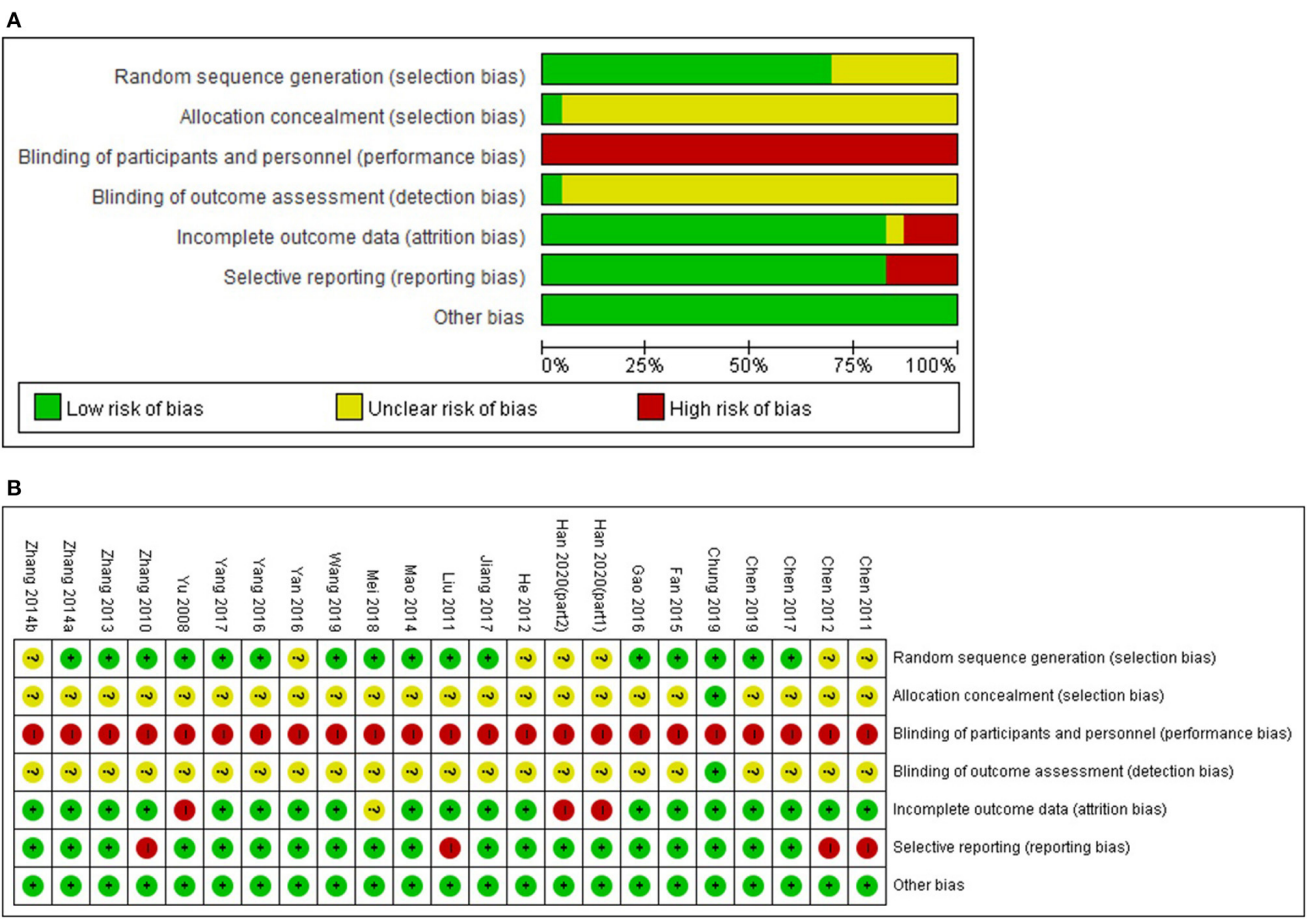
**(A)** Risk of bias graph, **(B)** Risk of bias summary. Low, unclear, and high risk, respectively, are represented with the following symbols: “+”, “?”, and “−”.

Sixteen studies using proper randomization method such as random number tables were evaluated as having a low risk of bias in the random sequence generation domain (24, 25, 27, 30, 31, 33, 34, 36–43, 45) and the remaining six studies were evaluated as having an unclear risk of bias because there was no mention of the randomization method (26, 28, 29, 32, 35, 44). There was no mention of allocation concealment, except for one study in which allocation concealment was performed using an opaque sealed envelope (42). All studies did not mention the blinding of participants and personnel. However, this was evaluated as having a high risk of bias in all studies due to all comparing the treatment and control interventions (acupuncture as add-on therapies to conventional WM vs. WM alone). As for the blinding of the outcome assessment, only one study mentioned that this was performed (42) while the other studies did not mention this. In two studies, dropouts occurred during the trial period, but the statistical analysis was performed using a per-protocol analysis method, and the risk of attrition bias was evaluated as high (24, 44). Four studies that presented only the TER without raw data were evaluated as having a high risk of reporting bias (25–28). All studies were evaluated as sufficiently homogeneous between the treatment and control groups in terms of the baseline characteristics (Figure 2).

### Effectiveness and Safety of Acupuncture as an Add-On Treatment for FD

#### Symptom Score (Primary Outcome)

A meta-analysis was not performed because of the heterogeneity of the symptom score scales used in the included studies. The results of each study are summarized in Table 3. In addition to the total score, ten individual symptoms including epigastric pain, epigastric burning, decreased food intake, postprandial fullness, belching, acid reflux, early satiation, nausea and/or vomiting, obstruction of the throat, and loss of appetite were evaluated. In one study that compared manual acupuncture combined with mosapride and mosapride alone, it was found that there was no significant difference in the symptom scores between the two groups (30). One study found that the total symptom score of manual acupuncture combined with domperidone and sucralfate was higher than the control group. However, they did not statistically analyze the score (24). All other studies found that the individual symptoms of FD were significantly improved in the acupuncture combined with the WM group compared to the WM alone group (29, 31–33, 35–37, 39–45).

**Table 3:** Symptom score and biomarkers related to functional dyspepsia.

**References**	**Study design**	**Total score**	**Epigastric pain**	**Epigastric burning**	**Decreased food intake**	**Postprandial fullness**	**Belching**	**Acid reflux**	**Early satiation**	**Nausea and/or vomiting**	**Obstruction of throat**	**Loss of appetite**	**Motilin**	**Ghrelin**	**5-HT**	**Gastrin**	**Somatostatin**
Chen et al. (37)	AA + prokinetics	(A) < (B)^*^	–	–	–	–	–	–	–	–	–	–	–	–	–	–	–
Chen et al. (41)	MA + prokinetics	–	(A) < (B)+	(A) < (B)+	–	(A) < (B)+	–	–	(A) < (B)+	–	–	–	(A) > (B)+	–	–	(A) > (B)+	–
Chung et al. (42)	EA + gastrocaine	–	(change value) (A) > (B)^*^	(change value) N.S	–	(change value) (A) > (B)+	–	–	(change value) (A) > (B)^*^	(change value) (A) > (B)^*^	–	–	–	–	–	–	–
Fan (33)	MA + prokinetics	–	(A) < (B)^*^	(A) < (B)^*^	–	(A) < (B)^*^	–	–	(A) < (B)^*^	–	–	–	–	–	–	–	–
Han (44)	EA + prokinetics + acid suppressants	(A) < (B)^*^	–	–	–	–	–	–	–	–	–	–	(A) > (B)^*^	–	–	–	–
Han (44)	EA + prokinetics + acid suppressants + antidepressants	(A) < (B)^*^	–	–	–	–	–	–	–	–	–	–	(A) > (B)^*^	–	–	–	–
He (29)	MA + prokinetics	(A) < (B)^*^	–	–	–	–	–	–	–	–	–	–	–	–	–	–	–
Mao (31)	EA + prokinetics	(A) < (B)^*^	(A) < (B)^*^	N.S	–	(A) < (B)^*^	–	–	(A) < (B)^*^	–	–	–	–	–	–	–	–
Mei (40)	EA + prokinetics	(A) < (B)^*^	–	–	–	–	–	–	–	–	–	–	–	–	–	–	–
Wang (43)	MA + prokinetics + acid suppressants	–	(A) < (B)+	(A) < (B)+	(A) < (B)+	(A) < (B)+	–	–	(A) < (B)+	–	–	–	–	–	–	–	–
Yan (35)	MA + prokinetics	–	(A) < (B)^*^	–	–	(A) < (B)^*^	–	–	(A) < (B)^*^	(A) < (B)^*^	–	(A) < (B)^*^	(A) > (B)^*^	(A) < (B)^*^	(A) < (B)^*^	–	–
Yang et al. (36)	MA + prokinetics + antidepressants	–	–	–	–	–	–	–	–	–	–	–	–	–	–	(A) < (B)+	–
Yang and Huang (39)	MA + prokinetics + antidepressants			–	–	–	–	–	–	–	–	–	(A) < (B)^*^	–	–	(A) < (B)^*^	(A) < (B)^*^
Yu (24)	MA + prokinetics + acid suppressants	(A) < (B) (no statistical data)	–	–	–	–	–	–	–	–	–	–	–	–	–	–	–
Zhang (30)	MA + prokinetics	–	N.S	–	N.S	N.S	N.S	N.S	N.S	N.S	N.S	–	–	–	–	–	–
Zhang (45)	MA + prokinetics	(A) < (B)^*^	–	–	–	–	–	–	–	–	–	–	–	–	–	–	–
Zhang (32)	MA + acid suppressants	–	(A) < (B)^*^	(A) < (B)^*^	–	–	(A) < (B)^*^	–	–	–	–	–	–	–	–	-	-

*AA, auricular acupuncture; EA, electro-acupuncture; MA, manual acupuncture; N.S, not significant; 5-HT, 5-Hydroxytryptamine*.

* *p < 0.05*,

*^+^p < 0.01, (A) treatment group, (B) control group*.

#### NDI Score (Primary Outcome)

Only one study reported the NDI scores (40). According to Mei (40), electroacupuncture combined with domperidone showed a significantly lower NDI score than the control group (1 study; MD −7.92, 95% CI −11.84 to −4.00) (Table 4) (40).

**Table 4:** Summary of findings and quality of evidence.

**Outcomes**		**No. participants (RCTs)**	**Anticipated absolute effects (95% CI)**		**Relative effect (95% CI)**	***I^**2**^* value**	**Quality of evidence (GRADE)**	**Comments**
			**Risk with control group**	**Risk with acupuncture group**				
NDI	Total (prokinetics; electro-acupuncture)	80 (1)	–	MD 7.92 lower (11.84–4 lower)	–	Not applicable	⊕⊕⊕○ MODERATE	Risk of bias (−1)
Total effective rate	Total	1,960 (20)	702 per 1,000	906 per 1,000 (864–941)	RR 1.29 (1.23–1.34)	14	⊕⊕○○ LOW	Risk of bias (−1) Indirectness (−1)
Subgroup 1	Prokinetics	1,076 (10)	741 per 1,000	926 per 1,000 (881–978)	RR 1.25 (1.19–1.32)	18	⊕⊕○○ LOW	Risk of bias (−1) Indirectness (−1)
	Acid suppressants	76 (1)	722 per 1,000	924 per 1,000 (744–1,000)	RR 1.28 (1.03–1.60)	Not applicable	⊕○○○ VERY LOW	Risk of bias (−1) Indirectness (−1) Imprecision (−1)
	Prokinetics + Acid suppressants	592 (7)	739 per 1,000	917 per 1,000 (850–983)	RR 1.24 (1.15–1.33)	0	⊕⊕○○ LOW	Risk of bias (−1) Indirectness (−1)
	Prokinetics + Acid suppressants + Antidepressants	84 (1)	780 per 1,000	976 per 1,000 (827–1,000)	RR 1.25 (1.06–1.48)	Not applicable	⊕○○○ VERY LOW	Risk of bias (−1) Indirectness (−1) Imprecision (−1)
	Gastrocaine	132 (1)	167 per 1,000	592 per 1,000 (332–1,000)	RR 3.55 (1.99–6.30)	Not applicable	⊕○○○ VERY LOW	Risk of bias (−1) Indirectness (−1) Imprecision (−1)
Subgroup 2	Manual acupuncture	1,495 (15)	755 per 1,000	936 per 1,000 (891–974)	RR 1.24 (1.18–1.29)	0	⊕⊕○○ LOW	Risk of bias (−1) Indirectness (−1)
	Electro-acupuncture	375 (4)	487 per 1,000	779 per 1,000 (667–910)	RR 1.60 (1.37–1.87)	85	⊕○○○ VERY LOW	Risk of bias (−1) Inconsistency (−2) Indirectness (−1)
	Auricular acupuncture	90 (1)	733 per 1,000	909 per 1,000 (748–1,000)	RR 1.24 (1.02–1.52)	Not applicable	⊕○○○ VERY LOW	Risk of bias (−1) Indirectness (−1) Imprecision (−1)
SF-36 (total)	Total (manual acupuncture)	190 (2)	–	MD 6.89 higher (5.32–8.47 higher)	–	86	⊕⊕⊕○ MODERATE	Risk of bias (−1)
	Prokinetics	90 (1)	–	MD 5.94 higher (4.22–7.66 higher)	–	Not applicable	⊕⊕⊕○ MODERATE	Risk of bias (−1)
	Prokinetics + Acid suppressants	100 (1)	–	MD 11.78 higher (7.87–15.69 higher)	–	Not applicable	⊕⊕⊕○ MODERATE	Risk of bias (−1)
SF-36 (vitality)	Total	180 (2)	–	MD 4.72 higher (2.57–6.87 higher)	–	72	⊕⊕⊕○ MODERATE	Risk of bias (−1)
	Prokinetics, Electro-acupuncture	80 (1)	–	MD 8.5 higher (4–13 higher)	–	Not applicable	⊕⊕⊕○ MODERATE	Risk of bias (−1)
	Prokinetics + Antidepressants, Manual acupuncture	100 (1)	–	MD 3.6 higher (1.15–6.05 higher)	–	Not applicable	⊕⊕⊕○ MODERATE	Risk of bias (−1)
SF-36 (physical functioning)	Total	180 (2)	–	MD 4.64 higher (1.64–7.64 higher)	–	0	⊕⊕⊕○ MODERATE	Risk of bias (−1)
	Prokinetics, Electro-acupuncture	80 (1)	–	MD 5.38 higher (0.45–10.31 higher)	–	Not applicable	⊕⊕⊕○ MODERATE	Risk of bias (−1)
	Prokinetics + Antidepressants, Manual acupuncture	100 (1)	–	MD 4.2 higher (0.42–7.98 higher)	–	Not applicable	⊕⊕⊕○ MODERATE	Risk of bias (−1)
SF-36 (bodily pain)	Total	180 (2)	–	MD 2.85 higher (0.4–5.3 higher)	–	18	⊕⊕⊕○ MODERATE	Risk of bias (−1)
	Prokinetics, Electro-acupuncture	80 (1)	–	MD 5.4 higher (0.27–10.53 higher)	–	Not applicable	⊕⊕⊕○ MODERATE	Risk of bias (−1)
	Prokinetics + Antidepressants, Manual acupuncture	100 (1)	–	MD 2.1 higher (0.68 lower−4.88 higher)	–	Not applicable	⊕⊕⊕○ LOW	Risk of bias (−1) Imprecision (−1)
SF-36 (general health perceptions)	Total	180 (2)	–	MD 3.74 higher (1.45–6.03 higher)	–	76	⊕○○○ VERY LOW	Risk of bias (−1) Inconsistency (−2)
	Prokinetics, Electro-acupuncture	80 (1)	–	MD 0.88 lower (5.84 lower−4.08 higher)	–	Not applicable	⊕⊕○○ LOW	Risk of bias (−1) Imprecision (−1)
	Prokinetics + Antidepressants, Manual acupuncture	100 (1)	–	MD 5 higher (2.41–7.59 higher)	–	Not applicable	⊕⊕⊕○ MODERATE	Risk of bias (−1)
SF-36 (physical role functioning)	Total	180 (2)	–	MD 3.23 higher (0.84–5.62 higher)	–	62	⊕⊕⊕○ MODERATE	Risk of bias (−1)
	Prokinetics, Electro-acupuncture	80 (1)	–	MD 9.38 higher (1.54–17.22 higher)	–	Not applicable	⊕⊕⊕○ MODERATE	Risk of bias (−1)
	Prokinetics + Antidepressants, Manual acupuncture	100 (1)	–	MD 2.6 higher (0.09–5.11 higher)	–	Not applicable	⊕⊕⊕○ MODERATE	Risk of bias (−1)
SF-36 (emotional role functioning)	Total	180 (2)	–	MD 3.34 higher (0.81–5.87 higher)	–	82	⊕⊕⊕○ MODERATE	Risk of bias (−1)
	Prokinetics, Electroacupuncture	80 (1)	–	MD 17.5 higher (5.36–29.64 higher)	–	Not applicable	⊕⊕⊕○ MODERATE	Risk of bias (−1)
	Prokinetics + Antidepressants, Manual acupuncture	100 (1)	–	MD 2.7 higher (0.11–5.29 higher)	–	Not applicable	⊕⊕⊕○ MODERATE	Risk of bias (−1)
SF-36 (social role functioning)	Total	180 (2)	–	MD 2.31 higher (0.22 lower−4.84 higher)	–	55	⊕○○○ VERY LOW	Risk of bias (−1) Inconsistency (−1) Imprecision (−1)
	Prokinetics, Electro-acupuncture	80 (1)	–	MD 6.87 higher (0.37–13.37 higher)	–	Not applicable	⊕⊕⊕○ MODERATE	Risk of bias (−1)
	Prokinetics + Antidepressants, Manual acupuncture	100 (1)	–	MD 1.5 higher (1.24 lower−4.24 higher)	–	Not applicable	⊕⊕○○ LOW	Risk of bias (−1) Imprecision (−1)
SF-36 (mental health)	Total	180 (2)	–	MD 8.36 higher (5.86–10.86 higher)	–	0	⊕⊕⊕○ MODERATE	Risk of bias (−1)
	Prokinetics, Electro-acupuncture	80 (1)	–	MD 11 higher (5.04–16.96 higher)	–	Not applicable	⊕⊕⊕○ MODERATE	Risk of bias (−1)
	Prokinetics + Antidepressants, Manual acupuncture	100 (1)	–	MD 7.8 higher (5.05–10.55 higher)	–	Not applicable	⊕⊕⊕○ MODERATE	Risk of bias (−1)
Incidence of adverse events	Total	1,209 (12)	65 per 1,000	73 per 1,000 (33–165)	RR 1.13 (0.50–2.54)	64	⊕○○○ VERY LOW	Risk of bias (−1) Inconsistency (−1) Imprecision (−1)
Subgroup 1	Prokinetics	738 (7)	76 per 1,000	71 per 1,000 (43–118)	RR 0.93 (0.56–1.55)	0	⊕⊕○○ LOW	Risk of bias (−1) Imprecision (−1)
	Acid suppressants	76 (1)	83 per 1,000	50 per 1,000 (9–283)	RR 0.60 (0.11–3.39)	Not applicable	⊕⊕○○ LOW	Risk of bias (−1) Imprecision (−1)
	Prokinetics + Acid suppressants	79 (1)	25 per 1,000	9 per 1,000 (0–204)	RR 0.34 (0.01–8.14)	Not applicable	⊕⊕○○ LOW	Risk of bias (−1) Imprecision (−1)
	Prokinetics + Antidepressants	100 (1)	0 per 1,000	0 per 1,000 (0–0)	Not estimable	Not applicable	⊕⊕⊕○ MODERATE	Risk of bias (−1)
	Prokinetics + Acid suppressants + Antidepressants	84 (1)	24 per 1,000	8 per 1,000 (0–185)	RR 0.32 (0.01–7.59)	Not applicable	⊕⊕○○ LOW	Risk of bias (−1) Imprecision (−1)
	Gastrocaine	132 (1)	91 per 1,000	621 per 1,000 (283–1,000)	RR 6.83 (3.11–14.99)	Not applicable	⊕⊕○○ LOW	Risk of bias (−1) Imprecision (−1)
Subgroup 2	Manual acupuncture	834 (8)	70 per 1,000	62 per 1,000 (38–104)	RR 0.89 (0.54–1.48)	0	⊕⊕○○ LOW	Risk of bias (−1) Imprecision (−1)
	Electro-acupuncture	375 (4)	53 per 1,000	76 per 1,000 (14–422)	RR 1.43 (0.26–7.90)	65	⊕○○○ VERY LOW	Risk of bias (−1) Inconsistency (−1) Imprecision (−1)
Recurrence rate	Total (manual acupuncture)	394 (3)	170 per 1,000	75 per 1,000 (43–129)	RR 0.44 (0.25–0.76)	0	⊕⊕○○ LOW	Risk of bias (−1) Imprecision (−1)
	Prokinetics	318 (2)	190 per 1,000	80 per 1,000 (44–142)	RR 0.42 (0.23–0.75)	0	⊕⊕○○ LOW	Risk of bias (−1) Imprecision (−1)
	Acid suppressants	76 (1)	83 per 1,000	50 per 1,000 (9–283)	RR 0.60 (0.11–3.39)	Not applicable	⊕⊕○○ LOW	Risk of bias (−1) Imprecision (−1)

*CI, confidence interval; MD, mean difference; NDI, Nepean dyspepsia index; RCT, randomized controlled trial; RR, risk ratio; SF-36, 36-item short-form health survey*.

#### TER (Secondary Outcome)

According to the meta-analysis, the acupuncture combined with WM group showed a significantly higher TER than the WM group (20 studies; RR 1.29, 95% CI 1.23–1.34; *I*^2^ = 14%). The superiority of acupuncture combined with WM remained significant in all subgroup analyses according to the WM type: (1) prokinetics (10 studies; RR 1.25, 95% CI 1.19–1.32; *I*^2^ = 18%); (2) acid suppressants (1 study; RR 1.28, 95% CI 1.03–1.60); (3) prokinetics and acid suppressants (seven studies; RR 1.24, 95% CI 1.15–1.33; *I*^2^ = 0%); (4) prokinetics, acid suppressants, and antidepressants (one study; RR 1.25, 95% CI 1.06–1.48); and (5) gastrocaine (one study; RR 3.55, 95% CI 1.99–6.30), as well as, according to acupuncture type: (1) manual acupuncture (15 studies; RR 1.24, 95% CI 1.18–1.29; *I*^2^ = 0%); (2) electroacupuncture (four studies; RR 1.60, 95% CI 1.37–1.87; *I*^2^ = 85%); and (3) auricular acupuncture (one study; RR 1.24, 95% CI 1.02–1.52) (Table 4, Supplementary File 4).

#### Short-Form Health Survey (Secondary Outcome)

Although SF-36 was used, one study with a different symptom score range was not included in the meta-analysis (30). Overall, the acupuncture combined with WM group showed significantly higher scores than the control group in terms of the total score (2 studies; MD 6.89, 95% CI 5.32–8.47; *I*^2^ = 86%) as well as almost SF-36 subscales including vitality (two studies; MD 4.72, 95% CI 2.57–6.87; *I*^2^ = 72%), physical functioning (two studies; MD 4.64, 95% CI 1.64–7.64; *I*^2^ = 0%), bodily pain (two studies; MD 2.85, 95% CI 0.40–5.30; *I*^2^ = 18%), general health perception (two studies; MD 3.74, 95% CI 1.45–6.03; *I*^2^ = 76%), physical role functioning (two studies; MD 3.23, 95% CI 0.84–5.62; *I*^2^ = 62%), emotional role functioning (two studies; MD 3.34, 95% CI 0.81–5.87; *I*^2^ = 82%), and mental health (two studies; MD 8.36, 95% CI 5.86–10.86; *I*^2^ = 0%), but not in social role functioning (two studies; MD 2.31, 95% CI −0.22 to 4.84; *I*^2^ = 55%). In the subscale of social role functioning, when a subgroup analysis was performed according to acupuncture type, manual acupuncture combined with WM showed no significant difference with the WM group (1 study; MD 1.50, 95% CI −1.24 to 4.24), but electroacupuncture combined with WM showed significantly superior results compared to the WM group (1 study; MD 6.87, 95% CI 0.37–13.37) (Table 4, Supplementary File 4).

#### Biomarkers Related to FD (Secondary Outcome)

A meta-analysis was not performed for the biomarkers related to FD because of the heterogeneity of the measurement unit. The most frequently measured biomarker was the serum motilin level, and four (35, 41, 44) out of five studies (35, 39, 41, 44) reported that the level was significantly higher in the acupuncture combined with WM group after treatment compared to the control group (*P* < 0.05, *P* < 0.01). In addition, other biomarkers such as ghrelin (35) 5-Hydroxytryptamine (5-HT) (35), gastrin (36, 39, 41) and somatostatin (39) were measured. However, their levels were measured only in a single study or showed inconsistent results (Table 3).

#### Safety Data (Secondary Outcome)

A total of 12 trials reported the safety profile of the interventions (27, 29–32, 36, 38, 41, 42, 44, 45). Generally, there was no significant difference in the incidence of adverse events between acupuncture combined with WM and WM alone (12 studies; RR 1.13, 95% CI 0.50–2.54; *I*^2^ = 64%). The only individual study that showed a statistically significant difference between the two groups was Chung et al. (42). This study reported that the incidence of adverse events was significantly higher in the acupuncture combined with the WM group (62.12 vs. 10.91%) (42). This difference was attributed to local pain, local bruising, and local numbness due to acupuncture stimulation. No severe adverse events were reported with respect to the interventions used (Table 4, Supplementary File 5).

#### Recurrence Rate (Secondary Outcome)

Three studies reported the recurrence rate of FD after the end of treatment (29, 32, 32). As a result, manual acupuncture combined with WM group had a significantly lower recurrence rate after 3–6 months of follow-up than the control group (three studies; RR 0.44, 95% CI 0.25–0.76; *I*^2^ = 0%). In the subgroup analysis according to WM type, the significant superiority remained in combination with prokinetics (two studies; RR 0.42, 95% CI 0.23–0.75; *I*^2^ = 0%), but not in combination with acid suppressants (one study; RR 0.60, 95% CI 0.11–3.39). All studies only used manual acupuncture (Table 4, Supplementary File 4).

### Sensitivity Analysis

In a meta-analysis of most outcomes other than TER, the number of studies was not sufficient to perform a sensitivity analysis excluding outliers. In the case of TER, even when an outlier (42) that was considered to be the main cause of statistical heterogeneity in the meta-analysis, was excluded from the sensitivity analysis, the existing results were not significantly affected (acupuncture combined with WM vs. WM alone, 19 studies, RR 1.25, 95% CI 1.20–1.30; *I*^2^ = 0%; and electroacupuncture combined WM vs. WM alone, three studies, RR 1.34, 95% CI 1.16–1.54; *I*^2^ = 39%). The results were similar in the sensitivity analysis, except for RCTs whose randomization method was unclear (28, 29, 32, 35, 44) or dissertation (24, 30, 31). The results of former were as follows: acupuncture combined with WM vs. WM alone (14 studies; RR 1.33, 95% CI 1.25–1.41; *I*^2^ = 57%), acupuncture combined with prokinetics vs. prokinetics alone (eight studies; RR 1.28, 95% CI 1.19–1.38; *I*^2^ = 35%), acupuncture combined with prokinetics and acid suppressants vs. prokinetics and acid suppressants (five studies; RR 1.25, 95% CI 1.15–1.36; *I*^2^ = 0%), manual acupuncture combined with WM vs. WM alone (11 studies; RR 1.25, 95% CI 1.17–1.32; *I*^2^ = 0%), and electroacupuncture combined with WM vs. WM alone (two studies; RR 2.31, 95% CI 1.71–3.13; *I*^2^ = 84%). The results of the latter were as follows: acupuncture combined with WM vs. WM alone (17 studies; RR 1.27, 95% CI 1.21–1.32; *I*^2^ = 31%), acupuncture combined with prokinetics vs. prokinetics alone (eight studies; RR 1.22, 95% CI 1.15–1.29; *I*^2^ = 0%), acupuncture combined with prokinetics and acid suppressants vs. prokinetics and acid suppressants (six studies; RR 1.23, 95% CI 1.13–1.33; *I*^2^ = 0%), manual acupuncture combined with WM vs. WM alone (13 studies; RR 1.22, 95% CI 1.17–1.28; *I*^2^ = 0%), and electroacupuncture combined with WM vs. WM alone (three studies; RR 1.58, 95% CI 1.33–1.88; *I*^2^ = 90%).

### Publication Bias

**Figure 3:**
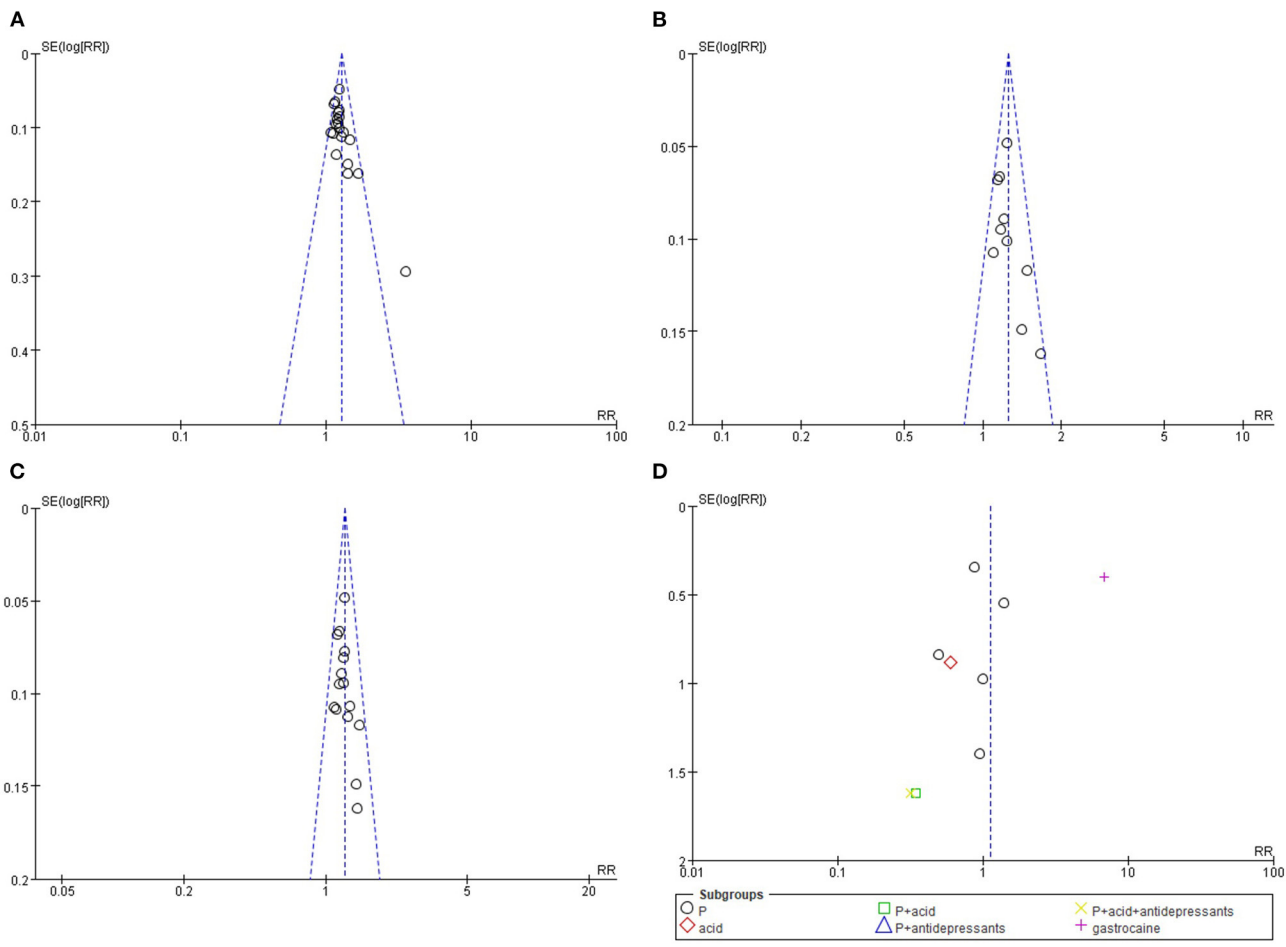
Funnel plots results. **(A)** total effective rate: acupuncture combined with Western medication vs. Western medication, **(B)** total effective rate: acupuncture combined with prokinetics vs. prokinetics, **(C)** total effective rate: manual acupuncture combined with Western medication vs. Western medication, **(D)** adverse events: acupuncture combined with Western medication vs. Western medication.

Four funnel plots were generated for the TER, adverse events, and secondary outcomes. Except for one outlier (42), which was excluded from the sensitivity analysis, no apparent asymmetry was observed overall. However, the funnel plot of the adverse events showed apparent asymmetry, suggesting a potential publication bias (Figure 3).

### QoE

The QoE of the NDI score, the primary outcome, was “moderate,” which was due to the high risk of bias of the included studies. The QoE for the TER ranged from “low” to “very low,” depending on the type of WM or the method of acupuncture used. This was due to the high risk of bias and the indirectness, inconsistency, and imprecision of the results. For SF-36, the QoE was generally evaluated as “moderate” or “low” due to the high risk of bias and imprecise results. Regarding the incidence of adverse events and recurrence rates, the QoE was generally low due to the high risk of bias and imprecise results (Table 4).

## Discussion

### Summary of Evidence

This systematic review was conducted to assess the effectiveness and safety of acupuncture as an add-on treatment to conventional WM for FD and critically evaluate the QoE. A total of 22 RCTs were included (24–45).

For the symptom scores, a meta-analysis was not performed due to the heterogeneity of the scales used. The studies included results for the total symptom score and ten individual FD symptom scores. Except for one study that did not find a significant difference between manual acupuncture combined with prokinetics and prokinetics alone in all evaluated symptom scores (30), the remaining studies supported the superiority of acupuncture combined with WM. The symptoms frequently improved with combined therapy were epigastric pain, epigastric burning, postprandial fullness, and early satiation. Only one study reported the NDI score, another primary outcome (40). In that study, electroacupuncture combined with domperidone showed significant superiority in terms of the NDI score after treatment compared to domperidone alone. In the secondary outcome TER, the most frequently reported outcome in the included studies, the acupuncture combined with WM group showed significantly higher TER than the WM group in both the meta-analysis and sensitivity analysis after removing outliers. Moreover, the superiority of acupuncture combined with WM remained significant in all subgroup analyses according to WM type (prokinetics, acid suppressants, prokinetics and acid suppressants, prokinetics and acid suppressants and antidepressants, and gastrocaine) as well as acupuncture type (manual acupuncture, electroacupuncture, and auricular acupuncture). In addition, the funnel plots did not suggest a potential publication bias. Acupuncture combined with WM showed superiority in most SF-36 results, but few studies have reported this outcome. As biomarkers related to FD, the levels of ghrelin, 5-HT, gastrin, and somatostatin showed mixed results, but motilin levels were significantly higher in the combined therapy group in most cases. Only three studies have reported the recurrence rate of FD after the end of treatment, and acupuncture combined with WM showed a significantly lower recurrence rate after 3–6 months of follow-up than WM alone. According to the subgroup analysis, it was particularly meaningful when combined with prokinetics but not with acid suppressants. Overall, acupuncture combined with WM did not show any significant difference in the incidence of adverse events compared to WM alone. However, the funnel plot suggests a potential publication bias.

The included studies had methodological flaws, especially those with a high risk of selection and performance bias. Most studies did not report proper allocation concealment or outcome assessment blinding procedures. In addition, no studies blinded the participants and personnel, suggesting potential performance bias. None of the studies were evaluated as having a low risk of bias in any of the seven bias items. In addition, the QoE evaluated by GRADE was often “moderate,” but there were still a number of studies evaluated as “low” or “very low.” No studies were rated as having high QoE. In particular, the QoE of TER, the most frequently reported outcome in the included studies, were all “very low” or “low.” This suggests that acupuncture combined with WM is likely to have potential therapeutic benefits in FD treatment, but the level of evidence is not high. Thus, interpretation of the findings requires caution.

### Comparison With Previous Studies

The findings of this review can be compared with those of some previous systematic reviews. Zhou et al. reported that acupuncture significantly improved FD symptoms as a monotherapy or add-on therapy and improved FD-related QoL as monotherapy but had no significant effect on plasma motilin with borderline significance (six studies; standardized mean difference 0.67, 95% CI −0.07 to 1.42; *I*^2^ = 95%) (10). However, our review focuses on acupuncture as an add-on treatment and emphasizes that there was insufficient evidence for QoL improvement with acupuncture. In addition, according to the presents findings, acupuncture as an add-on treatment is associated with a significant increase in plasma motilin levels; however, this finding may be influenced by the results of studies published after the systematic review by Zhou et al. In an overview of systematic reviews performed by Ho et al. using network meta-analysis (NMA), it was concluded that the combination of manual acupuncture and clebopride was most effective in alleviating FD symptoms (11). In this NMA, other acupuncture types such as electroacupuncture or auricular acupuncture were not considered as add-on therapies (11). However, the subgroup analysis of our review showed that we should continue considering acupuncture types other than manual acupuncture as add-on therapies. Guo et al. reported that acupuncture and electroacupuncture potentially help improve FD symptoms and QoL (12), which is consistent the present review findings; in particular, this review focused on the therapeutic mechanisms of acupuncture and electroacupuncture for FD and suggested the regulation of gastric motility, gastric accommodation, mental status, gastrointestinal hormones, and central and autonomic functions as one of the underlying mechanisms (12), which supports the findings of our review. Finally, Mao et al. focused on electroacupuncture as a monotherapy for FD and reported that the therapeutic effect of electroacupuncture on FD is equivalent to that of WM on FD (13). As a monotherapy, acupuncture can be suggested as a treatment option for FD; however, as an add-on therapy, which was the scope of our study, it can be considered as a promising treatment option for FD. The implementation of treatment options may be selected based on factors such as resources in clinical settings, symptoms of patients, values and preferences of patients, clinical evidence for the effectiveness and safety of treatment options, and cost-effectiveness, if possible. Given the above findings, further studies comparing acupuncture as a monotherapy and acupuncture combined with conventional medication for FD in FD treatment may be helpful in the practical application of acupuncture-based treatment options.

### Clinical Implications

FD is a common functional gastrointestinal disorder that causes impaired QoL in patients and socioeconomic burden (5, 6). Acupuncture, a non-pharmacological CAM treatment, has been reported to be effective and safe for the treatment of FD (10–13), and has the potential to improve the management of FD in combination with conventional WM. Acupuncture is also clinically useful because it is non-pharmacological and free of potential interactions with conventional WM.

Despite the studies not providing the best evidence, this review's findings generally suggest that acupuncture improves the symptoms of FD and some FD-related biomarkers when combined with conventional WM. In particular, in terms of TER, which has been reported frequently, acupuncture showed significant benefits in combination with prokinetics or acid suppressants. In addition, the therapeutic benefits of TER were maintained regardless of the type of acupuncture, namely manual acupuncture, electroacupuncture, and auricular acupuncture. The acupuncture method for FD showed inconsistency between the included studies, but ST36, PC6, and CV12 were the most frequently used acupoints, and De qi was generally performed. The treatment duration and number of sessions were the most common at four and 28 sessions, respectively. This was consistent with the results of the most frequently chosen acupoints in FD treatment in a recent survey by clinicians (47). In the acupuncture procedure, although stimulation for several acupoints produces the therapeutic effects in combination, the mechanism by which each acupoint stimulation contributes to the improvement of dyspepsia has also been reported. For example, improvement in dyspepsia symptoms associated with ST36 stimulation may be related to vagal and gastrointestinal hormonal mechanisms and inhibition of excessive autophagy of interstitial cells of Cajal (48, 49). There is clinical evidence that a combination of ST36 and PC6 stimulation improves gastric accommodation and gastric emptying in patients with FD (50, 51). One scholar also asserted that antinociceptive effects could be expected with ST36 and PC6 stimulation, while the inhibitory effect of gastric acid secretion through the somatosympathetic pathway can be expected with CV12 stimulation (52). In acupuncture, FD is usually treated using several acupoints. If the mechanism of stimulation of each acupoint is elucidated in more detail, it is expected that more effective treatment strategies can be established according to the subtype of FD and the predominant symptoms of patients.

### Strengths and Limitations

This systematic review evaluated the role of acupuncture as an add-on therapy for FD treatment in terms of EBM. A subgroup analysis according to conventional WM and acupuncture type was conducted to resolve potential heterogeneity. The QoE of the findings of this review was strictly evaluated using the GRADE approach.

However, the following limitations should be considered. First, all studies included in this systematic review were conducted in China, and their risk of bias was not low enough to be reliable. This suggests that the findings could be challenging to generalize, particularly in countries other than China. As a country that has been using acupuncture for a long time, the Chinese may have a more favorable attitude toward acupuncture than other ethnicities, potentially contributing to placebo effects. In particular, the studies included in this review may be more susceptible to this problem because they are flawed in terms of performance bias. This suggests that further research using sham acupuncture is required. Moreover, only studies conducted in China were included, which could be a source of potential publication bias (53). Therefore, further studies evaluating the effectiveness and safety of add-on acupuncture in FD treatment should be conducted in countries other than China. Second, the diversity of the acupuncture methods used in the included studies may have contributed to the heterogeneity of the findings. Although CAM treatment, such as acupuncture, may emphasize customized treatment according to the individual patient's characteristics, developing a basic treatment standard that allows for some modifications could improve the quality of clinical evidence in this field. Third, the outcome most often reported in the included studies was TER. Although the gold standard evaluation tool for FD has not been determined, it is necessary to use a validated evaluation scale for gastrointestinal symptoms. In addition, although FD is a condition that seriously impairs the QoL of patients, only a small number of the included studies evaluated the effect of acupuncture on QoL. Therefore, further studies should be conducted that evaluate patients' QoL. Finally, other outcomes that can be objectively evaluated, such as FD-related biomarkers or detection of gastric myoelectrical activity using electrogastrography, could also be undertaken.

## Conclusion

In combination with conventional WM, acupuncture may be able to improve the symptoms of patients with FD. However, the methodological quality of the included studies and the QoE of the main findings were generally low. In addition, all of the included studies were conducted in China, which makes generalization of the findings difficult and infers potential publication bias. Therefore, RCTs with a rigorous methodology, including sham acupuncture and multiethnic subjects, should be performed.

## Data Availability Statement

The original contributions presented in the study are included in the article/Supplementary Material, further inquiries can be directed to the corresponding author/s.

## Author Contributions

The study was conceptualized by S-JK and J-WP. The study search, study screening, data extraction, and quality assessment were conducted by C-YK and BL. The manuscript was drafted by C-YK and BL and revised by S-JK, J-WP, JY, and JC. All authors contributed to the article and approved the submitted version.

## Conflict of Interest

The authors declare that the research was conducted in the absence of any commercial or financial relationships that could be construed as a potential conflict of interest.

## Publisher's Note

All claims expressed in this article are solely those of the authors and do not necessarily represent those of their affiliated organizations, or those of the publisher, the editors and the reviewers. Any product that may be evaluated in this article, or claim that may be made by its manufacturer, is not guaranteed or endorsed by the publisher.

## Abbreviations

CAM: complementary and alternative medicine
CI: confidence interval
EBM: evidence-based medicine
EPS: epigastric pain syndrome
FD: functional dyspepsia
GRADE: Grading of Recommendations, Assessment, Development, and Evaluation
MD: mean difference
NDI: Nepean dyspepsia index
PDS: postprandial distress syndrome
PRISMA: Preferred Reporting Items for Systematic Reviews and Meta-Analyses
QoE: quality of evidence
QoL: quality of life
RCT: randomized controlled trial
RR: risk ratio
SF-36: 36-item short-form health survey
TER: total effective rate
WM: Western medication
5-HT: 5-Hydroxytryptamine.

## References

[1] Stanghellini V Chan FK Hasler WL Malagelada JR Suzuki H Tack J. Gastroduodenal disorders. Gastroenterology. (2016) 150:1380–92. 10.1053/j.gastro.2016.02.011 27147122

[2] Talley NJ Goodsall T Potter M. Functional dyspepsia. Aust Prescriber. (2017) 40:209–13. 10.18773/austprescr.2017.066 PMC5768602 29375182

[3] Madisch A Andresen V Enck P Labenz J Frieling T Schemann M. The diagnosis and treatment of functional dyspepsia. Deutsches Arzteblatt Int. (2018) 115:222–32. 10.3238/arztebl.2018.0222 PMC5938438 29669681

[4] Mahadeva S Ford AC. Clinical and epidemiological differences in functional dyspepsia between the East and the West. Neurogastroenterol Motil. (2016) 28:167–74. 10.1111/nmo.12657 26331919

[5] Brook RA Kleinman NL Choung RS Melkonian AK Smeeding JE Talley NJ. Functional dyspepsia impacts absenteeism and direct and indirect costs. Clin Gastroenterol Hepatol. (2010) 8:498–503. 10.1016/j.cgh.2010.03.003 20304102

[6] Lacy BE Weiser KT Kennedy AT Crowell MD Talley NJ. Functional dyspepsia: the economic impact to patients. Aliment Pharmacol Ther. (2013) 38:170–7. 10.1111/apt.12355 23725230

[7] Chiarioni G Pesce M Fantin A Sarnelli G. Complementary and alternative treatment in functional dyspepsia. United Eur Gastroenterol J. (2018) 6:5–12. 10.1177/2050640617724061 29435308 PMC5802680

[8] Miwa H Kusano M Arisawa T Oshima T Kato M Joh T. Evidence-based clinical practice guidelines for functional dyspepsia. J Gastroenterol. (2015) 50:125–39. 10.1007/s00535-014-1022-3 25586651

[9] Sayuk GS Gyawali CP. Functional dyspepsia: diagnostic and therapeutic approaches. Drugs. (2020) 80:1319–36. 10.1007/s40265-020-01362-4 32691294

[10] Zhou W Su J Zhang H. Efficacy and safety of acupuncture for the treatment of functional dyspepsia: meta-analysis. J Altern Complement Med. (2016) 22:380–9. 10.1089/acm.2014.0400 27028618

[11] Ho RST Chung VCH Wong CHL Wu JCY Wong SYS Wu IXY. Acupuncture and related therapies used as add-on or alternative to prokinetics for functional dyspepsia: overview of systematic reviews and network meta-analysis. Sci Rep. (2017) 7:10320. 10.1038/s41598-017-09856-0 28871092 PMC5583250

[12] Guo Y Wei W Chen JD. Effects and mechanisms of acupuncture and electroacupuncture for functional dyspepsia: a systematic review. World J Gastroenterol. (2020) 26:2440–57. 10.3748/wjg.v26.i19.2440 32476804 PMC7243644

[13] Mao X Guo S Ni W Zhang T Liu Q Du S. Electroacupuncture for the treatment of functional dyspepsia: a systematic review and meta-analysis. Medicine. (2020) 99:e23014. 10.1097/MD.0000000000023014 33157947 PMC7647594

[14] Kwon CY Ko SJ Lee B Cha JM Park JW. Acupuncture as add-on treatment for functional dyspepsia: a protocol for systematic review. Medicine. (2021) 100:e24403. 10.1097/MD.0000000000024403 33607774 PMC7899868

[15] Moher D Liberati A Tetzlaff J Altman DG. Preferred reporting items for systematic reviews and meta-analyses: the PRISMA statement. PLoS Med. (2009) 6:e1000097. 10.1371/journal.pmed.1000097 19621072 PMC2707599

[16] Talley NJ Haque M Wyeth JW Stace NH Tytgat GN Stanghellini V. Development of a new dyspepsia impact scale: the nepean dyspepsia index. Aliment Pharmacol Ther. (1999) 13:225–5. 10.1046/j.1365-2036.1999.00445.x 10102954

[17] Adam B Liebregts T Saadat-Gilani K Vinson B Holtmann G. Validation of the gastrointestinal symptom score for the assessment of symptoms in patients with functional dyspepsia. Aliment Pharmacol Ther. (2005) 22:357–63. 10.1111/j.1365-2036.2005.02572.x 16098003

[18] Leidy NK Farup C Rentz AM Ganoczy D Koch KL. Patient-based assessment in dyspepsia: development and validation of Dyspepsia Symptom Severity Index (DSSI). Dig Dis Sci. (2000) 45:1172–9. 10.1023/A:1005558204440 10877234

[19] Lee EH Hahm KB Lee JH Park JJ Lee DH Kim SK. Development and validation of a functional dyspepsia-related quality of life (FD-QOL) scale in South Korea. J Gastroenterol Hepatol. (2006) 21:268–74. 10.1111/j.1440-1746.2006.04196.x 16460485

[20] Ware JE Jr Sherbourne CD. The MOS 36-item short-form health survey (SF-36). I. Conceptual framework and item selection. Med Care. (1992) 30:473–83. 10.1097/00005650-199206000-00002 1593914

[21] Higgins JP Altman DG. The Cochrane Collaboration. Chapter 8: assessing risk of bias in included studies. In: Higgins JP Altman DG editors. Cochrane Handbook for Systematic Reviews of Interventions Version 5.1.0. (2011). Available online at: http://www.cochrane-handbook.org (accessed January, 2021).

[22] Guyatt G Rennie D Meade M Cook D. Users' *Guides* to the *Medical Literature: a Manual for Evidence-Based Clinical Practice*. AMA press Chicago (2002).

[23] Balshem H Helfand M Schunemann HJ Oxman AD Kunz R Brozek J. GRADE guidelines: 3. Rating the quality of evidence. J Clin Epidemiol. (2011) 64:401–6. 10.1016/j.jclinepi.2010.07.015 21208779

[24] Yu X. The Clinical Research of the Effects of Acupuncture Treatment for Functional Despepsia (FD) Patients With Depssion and Anxiety (Master's degree). Beijing: Capital Medical University (2008).

[25] Zhang K. Analysis of 61 cases of functional dyspepsia treated with integrated traditional Chinese and Western Medicine. Chin Commun Doctors. (2010) 12:120.

[26] Chen E Chen L Sun D Ma D. Combination of acupuncture and rabeprazole in the treatment of functional dyspepsia. Jilin J Trad Chin Med. (2011) 31:785−6. 10.13463/j.cnki.jlzyy.2011.08.036

[27] Liu C Shu J. Clinical curative effect observing of acupuncture combined with clebopride on functional dyspepsia. Med Innovat China. (2011) 8:3–4.

[28] Chen LD Chen EP Sun DZ Ma DS. Treatment of functional dyspepsia with acupuncture on Zusanli and Neiguan with lansoprazole. Med J West China. (2012) 24:545−6.

[29] He CL. Clinical efficacy of mosapride and acupuncture used in the treatment of FD. J Qiqihar Univ Med. (2012) 33:2906–7.

[30] Zhang X. Clinical Study on Abdominal Acupuncture Treatment of Functional Dyspepsia (Master's degree). Guangzhou: Guangzhou University of Chinese Medicine (2013).

[31] Mao Y. Clinical Observation on Electro Acupuncture Combined With Mosapride in the Treatment on Type of Liver-Stomach Disharmony of Functional Dyspepsia (Master's degree). Wuhan: Hubei University of Chinese Medicine (2014).

[32] Zhang M. Study on the therapeutic effect of rabeprazole combined with traditional Chinese medicine acupuncture and moxibustion on functional dyspepsia. Contemp Med. (2014) 20:158–9. 10.3969/j.issn.1009-4393.2014.16.112

[33] Fan Z. Clinical observation on 56 cases of functional dyspepsia treated by acupuncture based on differentiation of symptoms and symptoms. J Gansu Coll Trad Chin Med. (2015) 32:61−3.

[34] Gao Y. Effect of acupuncture at Neiguan and Zusanli on the curative effect of patients with functional dyspepsia. Clin Res. (2016) 24:177−8.

[35] Yan Z. Clinical observation of Mosapride Citrate Tablets combined with acupuncture on the treatment of functional dyspepsia. Hebei J TCM. (2016) 38:1046−50. 10.3969/j.issn.1002-2619.2016.07.023

[36] Yang Z Wang J An J Liu L Cheng Y Jia X. Analysis of the effect of Laoshizhen on functional dyspepsia with sleep disturbance. J Clin Acupunct Moxibust. (2016) 32:23−6.

[37] Chen Y Bi D Zhu W Luo J. The effect on press-needle combined with mosapride in the treatment of elderly patients with functional dyspepsia. J Zhejiang Chin Med Univ. (2017) 41:911−4. 10.16466/j.issn1005-5509.2017.11.017

[38] Jiang GD. Clinical curative effect and EGG changes of functional dyspepsia treated by acupuncture combined with medicine. JCAM. (2017) 33:20−3.

[39] Yang H Huang H. Effect of hewei anshen acupuncture on gastrointestinal function and quality of life in patients with functional dyspepsia and sleep disorder. Guiding J Trad Chin Med Pharmacol. (2017) 23:95−7. 10.13862/j.cnki.cn43-1446/r.2017.12.032

[40] Mei J. Curative effect observation of dialectical acupuncture in the treatment of functional dyspepsia. CJCM. (2018) 10:95−6. 10.3969/j.issn.1674-7860.2018.36.042

[41] Chen F Mu Y Xu T Wu X. Buqi xiaopi acupuncture method in treating functional dyspepsia and effect on serum gastric hormones. J Shandong Univ TCM. (2019) 43:577–80. 10.16294/j.cnki.1007-659x.2019.06.011

[42] Chung VCH Wong CHL Wu IXY Ching JYL Cheung WKW Yip BHK. Electroacupuncture plus on-demand gastrocaine for refractory functional dyspepsia: pragmatic randomized trial. J Gastroenterol Hepatol. (2019) 34:2077–85. 10.1111/jgh.14737 31117149

[43] Wang G. Clinical observation on abdominal acupuncture in the treatment of functional dyspepsia with liver depression and spleen deficiency. CJGMCM. (2019) 34:1229–31. 10.3969/j.issn.1003-8914.2019.08.037

[44] Han SL Chen XY. Clinical effect of agomeladine, acupuncture combined with conventional western medical therapy on menopusal patient with functional dypepsia. Henan Med Res. (2020) 29:2539−42. 10.3969/j.issn.1004-437X.2020.14.014

[45] Zhang B Zhang P. Treatment of 30 cases of functional dyspepsia of liver qi invading stomach by acupuncture and medicine. Fujian J Trad Chin Med. (2014) 45:40–1. 10.13260/j.cnki.jfjtcm.010712

[46] Zhang M. Study on the therapeutic effect of rabeprazole combined with traditional Chinese medicine acupuncture and moxibustion on functional dyspepsia. Contemp Med. (2014) 20:158–9.

[47] Kim SY Hong SH Park JW Lee H Kim J Kim Y. Analysis of diagnostic decision in acupuncture from the actual functional dyspepsia patient's clinical information. Integr Med Res. (2020) 9:100419. 10.1016/j.imr.2020.100419 32455110 PMC7236055

[48] Liu Y Zhang S Ye F Yin J Li S Chen JDZ. Ameliorating effects and mechanisms of chronic electroacupuncture at ST36 in a rodent model of dyspepsia induced by cisplatin. Neurogastroenterol Motil. (2019) 31:e13474. 10.1111/nmo.13474 30246392

[49] Pan XL Zhou L Wang D Han YL Wang JY Xu PD. [Electroacupuncture at “Zusanli”(ST36) promotes gastrointestinal motility possibly by suppres-sing excessive autophagy via AMPK/ULK1 signaling in rats with functional dyspepsia]. Zhen Ci Yan Jiu. (2019) 44:486−91. 10.13702/j.1000-0607.180571 31368278

[50] Xu S Hou X Zha H Gao Z Zhang Y Chen JD. Electroacupuncture accelerates solid gastric emptying and improves dyspeptic symptoms in patients with functional dyspepsia. Dig Dis Sci. (2006) 51:2154–9. 10.1007/s10620-006-9412-x 17082991

[51] Xu F Tan Y Huang Z Zhang N Xu Y Yin J. Ameliorating effect of transcutaneous electroacupuncture on impaired gastric accommodation in patients with postprandial distress syndrome-predominant functional dyspepsia: a pilot study. Evid Based Complement Alternat Med. (2015) 2015:168252. 10.1155/2015/168252 26064155 PMC4433673

[52] Takahashi T. Acupuncture for functional gastrointestinal disorders. J Gastroenterol. (2006) 41:408–17. 10.1007/s00535-006-1773-6 16799881

[53] Vickers A Goyal N Harland R Rees R. Do certain countries produce only positive results? A systematic review of controlled trials. Control Clin Trials. (1998) 19:159–66. 10.1016/S0197-2456(97)00150-5 9551280

