# Supplementary material for: Acupuncture as an Add-On Treatment for Functional Dyspepsia: A Systematic Review and Meta-Analysis
Source: Front Med (Lausanne). 2021 Jul 26;8:682783. doi: 10.3389/fmed.2021.682783 (PMC8350114; doi:10.3389/fmed.2021.682783)
Supplement: Supplementary file 1 [file Data_Sheet_1.pdf]

Supplement 1. PRISMA 2010 checklist

Section/topic	#	Checklist item	Reported on page #
**TITLE**			
Title	1	Identify the report as a systematic review, meta-analysis, or both.	1
**ABSTRACT**			
Structured summary	2	Provide a structured summary including, as applicable: background; objectives; data sources; study eligibility criteria, participants, and interventions; study appraisal and synthesis methods; results; limitations; conclusions and implications of key findings; systematic review registration number.	2
**INTRODUCTION**			
Rationale	3	Describe the rationale for the review in the context of what is already known.	3
Objectives	4	Provide an explicit statement of questions being addressed with reference to participants, interventions, comparisons, outcomes, and study design (PICOS).	3
**METHODS**			
Protocol and registration	5	Indicate if a review protocol exists, if and where it can be accessed (e.g., Web address), and, if available, provide registration information including registration number.	4
Eligibility criteria	6	Specify study characteristics (e.g., PICOS, length of follow-up) and report characteristics (e.g., years considered, language, publication status) used as criteria for eligibility, giving rationale.	4
Information sources	7	Describe all information sources (e.g., databases with dates of coverage, contact with study authors to identify additional studies) in the search and date last searched.	4
Search	8	Present full electronic search strategy for at least one database, including any limits used, such that it could be repeated.	Supplement 2

Study selection	9	State the process for selecting studies (i.e., screening, eligibility, included in systematic review, and, if applicable, included in the meta-analysis).	5
Data collection process	10	Describe method of data extraction from reports (e.g., piloted forms, independently, in duplicate) and any processes for obtaining and confirming data from investigators.	5
Data items	11	List and define all variables for which data were sought (e.g., PICOS, funding sources) and any assumptions and simplifications made.	5
Risk of bias in individual studies	12	Describe methods used for assessing risk of bias of individual studies (including specification of whether this was done at the study or outcome level), and how this information is to be used in any data synthesis.	5
Summary measures	13	State the principal summary measures (e.g., risk ratio, difference in means).	5
Synthesis of results	14	Describe the methods of handling data and combining results of studies, if done, including measures of consistency (e.g., I^2) for each meta-analysis.	5-6

Section/topic	#	Checklist item	Reported on page #
Risk of bias across studies	15	Specify any assessment of risk of bias that may affect the cumulative evidence (e.g., publication bias, selective reporting within studies).	6
Additional analyses	16	Describe methods of additional analyses (e.g., sensitivity or subgroup analyses, meta-regression), if done, indicating which were pre-specified.	5-6
**RESULTS**			
Study selection	17	Give numbers of studies screened, assessed for eligibility, and included in the review, with reasons for exclusions at each stage, ideally with a flow diagram.	7<br>Figure 1

Study characteristics	18	For each study, present characteristics for which data were extracted (e.g., study size, PICOS, follow-up period) and provide the citations.	7-8
Risk of bias within studies	19	Present data on risk of bias of each study and, if available, any outcome level assessment (see item 12).	8
Results of individual studies	20	For all outcomes considered (benefits or harms), present, for each study: (a) simple summary data for each intervention group (b) effect estimates and confidence intervals, ideally with a forest plot.	8-10
Synthesis of results	21	Present results of each meta-analysis done, including confidence intervals and measures of consistency.	9-10
Risk of bias across studies	22	Present results of any assessment of risk of bias across studies (see Item 15).	11<br>Figure 3
Additional analysis	23	Give results of additional analyses, if done (e.g., sensitivity or subgroup analyses, meta-regression [see Item 16]).	10-11
**DISCUSSION**			
Summary of evidence	24	Summarize the main findings including the strength of evidence for each main outcome; consider their relevance to key groups (e.g., healthcare providers, users, and policy makers).	12
Limitations	25	Discuss limitations at study and outcome level (e.g., risk of bias), and at review-level (e.g., incomplete retrieval of identified research, reporting bias).	14-15
Conclusions	26	Provide a general interpretation of the results in the context of other evidence, and implications for future research.	15
**FUNDING**			
Funding	27	Describe sources of funding for the systematic review and other support (e.g., supply of data); role of funders for the systematic review.	15

From: Moher D, Liberati A, Tetzlaff J, Altman DG, The PRISMA Group (2009). Preferred Reporting Items for Systematic Reviews and Meta-Analyses: The PRISMA Statement. PLoS Med

6(7): e1000097. doi:10.1371/journal.pmed1000097

For more information, visit: **www.prisma-statement.org**.

Supplement 2. Search strategies used in each database and results

Medline via PubMed

	Searches	Results
#1	indigestion*	1073
#2	intestin* OR digest* OR gastr* OR gut OR epigastr* OR stomach*	2121855
#3	#1 AND #2	641
#4	dyspepsia*	14150
#5	epigastric[TIAB] AND pain[TIAB]	5988
#6	epigastric[TIAB] AND burn*[TIAB]	299
#7	Rome* AND criteria*	9415
#8	(disturbance* OR disorder* OR difficult* OR dysfunction* OR disease* OR impair* OR condition* OR abnormal* OR illness* OR patholog* OR discomfort* OR hazard* OR damage* OR injur* OR irritab* OR pain* OR distress* OR burning) AND postprandial*	15202
#9	#3 OR #4 OR #5 OR #6 OR #7 OR #8	42921
#10	Acupuncture[MH] OR "Acupuncture Therapy"[MH] OR "Acupuncture Points"[MH] OR acupunct*[TIAB] OR Electroacupuncture[MH] OR electroacupunct*[TIAB] or electro-acupunct*[TIAB] OR Auriculotherapy[MH] OR "ear acupuncture"[TIAB] OR acupoint[TIAB] OR Acupressure[MH] OR acupressure[TIAB]	32969
#11	"Randomized Controlled Trial"[PT] OR "Controlled Clinical Trial"[PT] OR randomized[TIAB] OR placebo[TIAB] OR "Clinical Trials as Topic"[Mesh: noexp] OR randomly[TIAB] OR trial[TI]	1419964
#12	animals[MH] NOT humans[MH]	4768626
#13	#9 AND #10 AND #11 NOT #12	**103**

EMBASE via Elsevier

	Searches	Results
#1	indigestion*	3505
#2	intestin* OR digest* OR gastr* OR gut OR epigastr* OR stomach*	2777248

#3	#1 AND #2	2122
#4	dyspepsia*	38587
#5	epigastric* AND pain*	24831
#6	epigastric* AND burn*	1348
#7	Rome* AND criteria*	23888
#8	(disturbance* OR disorder* OR difficult* OR dysfunction* OR disease* OR impair* OR condition* OR abnormal* OR illness* OR patholog* OR discomfort* OR hazard* OR damage* OR injur* OR irritab* OR pain* OR distress* OR burning) AND postprandial*	22975
#9	#3 OR #4 OR #5 OR #6 OR #7 OR #8	105913
#10	Acupuncture/exp OR acupuncture OR ‘acupuncture point’/exp OR ‘acupuncture point’ OR acupoint OR electroacupuncture/exp OR electroacupuncture OR auriculotherapy OR ‘auricular acupuncture’/exp OR ‘auricular acupuncture’ OR ‘ear acupuncture’ OR acupressure/exp OR acupressure	55823
#11	'randomized controlled trial'/exp OR 'randomized controlled trial'	840234
#12	'controlled clinical trial'/exp OR 'controlled clinical trial'	822826
#13	'placebo'/exp OR placebo	471570
#14	#11 OR #12 OR #13	1293328
#15	#9 AND #10 AND #14	**223**

CENTRAL

	Searches	Results
#1	(indigestion* AND (intestin* OR digest* OR gastr* OR gut OR epigastr* OR stomach*)):ti,ab,kw	292
#2	(dyspepsia* OR (epigastric AND pain) OR (epigastric AND burn*) OR (Rome* AND criteria*)):ti,ab,kw	7965
#3	((disturbance* OR disorder* OR difficult* OR dysfunction* OR disease* OR impair* OR condition* OR abnormal* OR illness* OR patholog* OR discomfort* OR hazard*	5567

	OR damage* OR injur* OR irritab* OR pain* OR distress* OR burning) AND postprandial*):ti,ab,kw	
#4	#1 OR #2 OR #3	13355
#5	MeSH descriptor: [Acupuncture] explode all trees	151
#6	MeSH descriptor: [Acupuncture Therapy] explode all trees	4749
#7	MeSH descriptor: [Acupuncture Points] explode all trees	2002
#8	MeSH descriptor: [Electroacupuncture] explode all trees	803
#9	MeSH descriptor: [Auriculotherapy] explode all trees	224
#10	MeSH descriptor: [Acupressure] explode all trees	356
#11	acupunct* OR electroacupunct* or electro-acupunct* OR "ear acupuncture" OR acupoint OR acupressure	17746
#12	#5 OR #6 OR #7 OR #8 OR #9 OR #10 OR #11	17863
#13	(#4 AND #12) in Trials	**200**

AMED via EBSCO

	Searches	Results
#1	indigestion*[TX]	43
#2	intestin*[TX] OR digest*[TX] OR gastr*[TX] OR gut[TX] OR epigastr*[TX] OR stomach*[TX]	7234
#3	#1 AND #2	25
#4	dyspepsia*[TX]	163
#5	epigastric[TX] AND pain[TX]	31
#6	epigastric[TX] AND burn*[TX]	3
#7	Rome*[TX] AND criteria*[TX]	26
#8	(disturbance*[TX] OR disorder*[TX] OR difficult*[TX] OR dysfunction*[TX] OR disease*[TX] OR impair*[TX] OR condition*[TX] OR abnormal*[TX] OR illness*[TX] OR patholog*[TX] OR discomfort*[TX] OR hazard*[TX] OR damage*[TX] OR injur*[TX] OR irritab*[TX] OR pain*[TX] OR distress*[TX] OR	61

	burning[TX]) AND postprandial*[TX]	
#9	#3 OR #4 OR #5 OR #6 OR #7 OR #8	283
#10	Acupuncture[SU] OR “Acupuncture Therapy”[SU] OR “Acupuncture Points”[SU] OR acupunct*[TX] OR Electroacupuncture[SU] OR electroacupunct*[TX] or electro-acupunct*[TX] OR Auriculotherapy[SU] OR “ear acupuncture”[TX] OR acupoint[TX] OR Acupressure[SU] OR acupressure[TX]	11928
#11	#9 AND #10	**38**

CINAHL via EBSCO

	Searches	Results
#1	indigestion*[TX]	2378
#2	intestin*[TX] OR digest*[TX] OR gastr*[TX] OR gut[TX] OR epigastr*[TX] OR stomach*[TX]	355994
#3	#1 AND #2	1653
#4	dyspepsia*[TX]	4656
#5	epigastric[TX] AND pain[TX]	2370
#6	epigastric[TX] AND burn*[TX]	456
#7	Rome*[TX] AND criteria*[TX]	8722
#8	(disturbance*[TX] OR disorder*[TX] OR difficult*[TX] OR dysfunction*[TX] OR disease*[TX] OR impair*[TX] OR condition*[TX] OR abnormal*[TX] OR illness*[TX] OR patholog*[TX] OR discomfort*[TX] OR hazard*[TX] OR damage*[TX] OR injur*[TX] OR irritab*[TX] OR pain*[TX] OR distress*[TX] OR burning[TX]) AND postprandial*[TX]	5636
#9	#3 OR #4 OR #5 OR #6 OR #7 OR #8	21708
#10	Acupuncture[MH] OR “Acupuncture Therapy”[MH] OR “Acupuncture Points”[MH] OR acupunct*[TX] OR Electroacupuncture[MH] OR electroacupunct*[TX] OR electro-acupunct*[TX] OR Auriculotherapy[MH] OR “ear acupuncture”[TX] OR acupoint[TX] OR Acupressure[MH] OR acupressure[TX]	34377
#11	#9 AND #10	**707**

OASIS

	Searches	Results
#1	기능성 소화불량 AND 침	**8**

KISS

	Searches	Results
#1	기능성 소화불량 AND 침	**9**

RISS

	Searches	Results
#1	기능성 소화불량 AND 침	**0**

KMBASE

	Searches	Results
#1	기능성 소화불량 AND 침	**5**

Supplement 3. Excluded studies after full-text review

I. Impossible to acquire full-text: 3

1. Therapeutic Effect of Acupuncture on Functional Dyspepsia Investigated by Electrogastrograpy. Abstracts of Academic Conference of the Tenth Anniversary of World Federation of Acupuncture-Moxibustion; 1997; 中国北京.
2. Cittadini M, Marmori F, Diacinti D, Walker JI. Randomized trial of acupuncture compared with prokinetic drugs and sham acupuncture for chronic idiopathic dyspepsia. *Med acup.* 2003;14(2):17-19.
3. Jin Y. Efficacy of acupuncture in the treatment of patients with functional dyspepsia. *Unpublished medicine master dissertation. Beijing university of chinese medicine.* 2011.

II. Study protocol: 1

1. Chi CI. Electroacupuncture plus standard care for managing refractory functional dyspepsia: pragmatic randomized trial with economic evaluation. *http://www.who.int/trialsearch/Trial2.aspx?TrialID=ChiCTR-IPC-15007109.* 2015.

III. Not clinical study: 3

1. 范正鹏. 针刺治疗功能性消化不良的脑功能研究现状. Paper presented at: 中国针灸学会临床分会2014年年会暨第二十一次全国针灸临床学术研讨会2014; 中国重庆.
2. 陈桂杰, 特木其勒, 红梅. 西沙必利联合蒙医温针治疗功能性消化不良的临床疗效观察. 2016.
3. 陈桂萍, 郑智红. 中西医结合治疗功能性消化不良浅探. *中外医疗.* 2010;29(12):188-188.

IV. Not RCT: 11

1. 针刺足三里对功能性消化不良患者血浆胃肠激素的影响. *基础医学与临床.* 2001(S1):68.
2. 针刺足三里对功能性消化不良患者胃运动总功率的影响. *基础医学与临床.* 2001(S1):67-68.
3. 郭钦源. 针刺治疗功能性消化不良60例临床疗效观察. *按摩与康复医学（上旬刊）.* 2011;02(6):50-51.
4. 林佳, 刘小琼. 针刺及手法治疗功能性消化不良71例. *中医药临床杂志.* 2016;28(08):1176-1178.
5. 斯亚琴. 针药结合治疗功能性消化不良98例. *浙江中西医结合杂志.* 2008(10):638-639.
6. 徐坚勇. 针刺足三里、内关、上巨虚对功能性消化不良患者疗效的影响. *东方食疗与保健.* 2016(11):206.
7. 徐世芳. 温针灸辅助治疗功能性消化不良的效果观察. *内蒙古中医药.* 2017;36(18):104-105.
8. 孙杨. 针刺治疗功能性消化不良32例. *中国针灸.* 2005;25(S1):64.
9. 余秀才. 针药结合治疗脾虚型功能性消化不良的疗效观察. *内蒙古中医药.* 2014;33(17):24-

25.

10. 周晶冰. 针灸治疗功能性消化不良(FD)伴随情绪障碍患者的临床疗效. *世界最新医学信息文摘(连续型电子期刊)*. 2020;20(5):99,101.
11. 秦晓勇, 孙国芳, 华小宁, 詹曦菁. 针刺治疗功能性消化不良症的疗效观察. *现代中西医结合杂志*. 2001(19):1883.

V. Not using acupuncture: 1

1. 王欢. 舒肝解郁胶囊治疗伴轻中度抑郁症状的 功能性消化不良患者的临床疗效及不良反应. *中国处方药*. 2019;17(5):91-92.

VI. Used acupuncture only: 12

1. Tang S, Xu Z, Tang P, Liang L. Acupuncture for functional dyspepsia: a randomised controlled trial. *Journal of sichuan traditional chinese medicine.* 2006;24(4):101-102.
2. Yuan XX, Wang BY, Yang L, Zhang YL. Clinical observation on acupuncture at Gongsun and Neiguan Points for functional dyspepsia patients with psychological factors. *Journal of clinical acupuncture and moxibustion [zhen jiu lin chuang za zhi].* 2015;31(4):52-55.
3. Zhou Y, Zhen JG. [Clinical Observations on Acupuncture Treatment of Functional Dyspepsia] (in Chinese). *Shanghai journal of acupuncture and moxibustion.* 2004;23(7):16-18.
4. 高用琨, 代二庆. 中医针灸治疗功能性消化不良的临床疗效. *临床医药文献电子杂志*. 2019;6(16):27-28.
5. 刘会堂, 顾兴江, 谭善瑞. 针灸治疗功能性消化不良疗效观察. *医学文选*. 1999;18(5):724.
6. 林琳. 针灸治疗功能性消化不良的临床疗效观察. *健康必读*. 2020(13):107.
7. 余梅. 针灸治疗功能性消化不良伴情绪障碍的临床研究. *养生保健指南*. 2019(9):237.
8. 王通兰, 唐先秀. 针刺穴位治疗功能性消化不良疗效观察. *中国保健营养*. 2018;28(23):259.
9. 张馨文, 拓西平. 指针疗法治疗功能性消化不良的临床研究. 第七届全国老年医学学术会议暨海内外华人老年医学学术会议论文汇编; 2004; 中国海口.
10. 钱拉拉, 张红, 金捷. 针刺对功能性消化不良患者近端胃容受功能及胃排空功能的影响. *上海针灸杂志*. 2017;36(09):1049-1052.
11. 赵航, 陈剑莉, 郑建寅, 管艳艳, 于洪涛, 王悦. 指针疗法治疗功能性消化不良的临床观察. 中国中西医结合学会第十四次全国消化系统疾病学术研讨会论文汇编; 2002; 中国杭州.
12. 崔延超, 杨运宽. 耳穴压丸加西药治疗功能性消化不良40例. *针灸临床杂志*. 2007;23(4):46-47.

VII. Combined with other TCM treatment: 13

1. Shi H, Zhang J, Guo H. Clinical observation of acupuncture for 90 cases of patients with functional dyspepsia. *Beijing journal of traditional chinese medicine.* 2009;28(9):732-733.

2. Zhou Y, Huang L. Effect of Abdominal Acupuncture Therapy on Quality of Life of Patients with Functional Dyspepsia. *Hubei Journal of Traditional Chinese Medicine.* 2011;33(1):38-39.
3. 段不屈. 针灸治疗功能性消化不良的临床疗效分析. *饮食保健.* 2019;6(42):75-76.
4. 李晓宇. 中医辨证疗法治疗脾胃气虚型功能性消化不良的可行性研究. *中医临床研究.* 2016;8(36):64-65.
5. 孙靖若. 健脾疏肝祛湿法加温针灸治疗功能性消化不良52例临床疗效观察. *按摩与康复医学.* 2012;3(009):201.
6. 时昭红, 胡畔, 石拓, 舒磊. 中西医结合治疗功能性消化不良脾胃湿热型的临床观察. 中华中医药学会脾胃病分会第二十四次全国脾胃病学术交流会论文集; 2012; 昆明.
7. 王宝立. 温针灸对脾胃气虚型功能性消化不良的临床效果分析. *中国社区医师.* 2015;31(24):89-90.
8. 王新亮. 针灸治疗功能性消化不良伴情绪障碍疗效观察. *中国实用医药.* 2018;13(25):30-31.
9. 熊明. 脉冲针灸治疗仪、多潘立酮联合治疗功能性消化不良的疗效观察. *中国中西医结合消化杂志.* 2012;20(5):227-228.
10. 张瑀. 针灸治疗功能性消化不良疗效观察. *中医临床研究.* 2018;10(29):44-45.
11. 曾令奉, 庹玲. 针灸对功能性消化不良伴睡眠障碍的应用价值. *辽宁中医杂志.* 2017;44(02):359-361.
12. 陈姗, 罗莎, 司莉莉, 李爱香. 中西医结合治疗老年功能性消化不良临床观察. *中国民族民间医药.* 2018;27(18):83-85.
13. 彭坤明, 罗鹏. 针灸治疗功能性消化不良伴情绪障碍疗效观察. *针灸临床杂志.* 2016;32(06):1-4.

VIII. Not using WM: 1

1. 佘文艳. 针对性护理干预对功能性消化不良患者症状及睡眠质量的影响观察. *基层医学论坛.* 2018;22(14):1991-1992.

IX. No details of WM: 2

1. 刘洪, 李慧臻, 刘琳. 足三里电针治疗功能性消化不良疗效分析. *中国中医药咨讯.* 2011;03(19):296.
2. 张奕秉, 金捷, 周乐盈, et al. 穴位针刺治疗功能性消化不良患者胃动力和感觉功能的作用机制研究. *中华物理医学与康复杂志.* 2019(08):616-618.

X. Compared two different TCMs: 2

1. Lima FAR, Ferreira LEVVC, Pace FHL. Acupuncture effectiveness as a complementary therapy in functional dyspepsia patients. *Arquivos de Gastroenterologia.* 2013;50(3):202-207.
2. 王成伟, 刘梦阅, 闫江华, et al. 针灸对伴情绪障碍功能性消化不良患者的疗效观察. *中国*

针灸. 2015;35(09):876-880.

XI. Compared two different WM: 1

1. 吴永华. 普瑞博思、针灸治疗老年运动障碍型、功能性消化不良. *中国社区医师*. 2002;18(5):35-35.

Supplement 4. Forest plots of meta-analyses in this review

1) TER (subgroup analysis according to WM type)

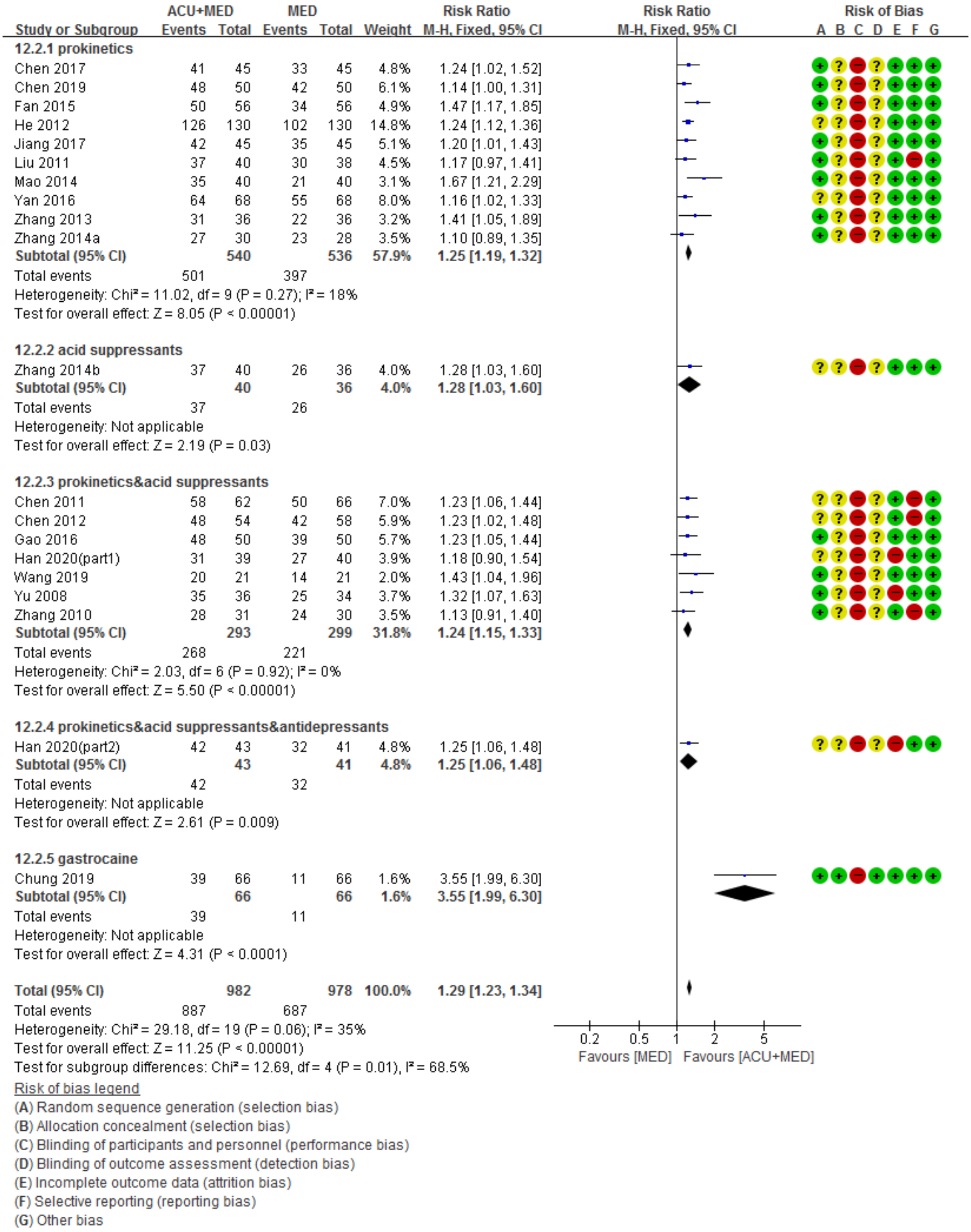

2) TER (subgroup analysis according to acupuncture type)

Study or Subgroup	ACU+MED Events	ACU+MED Total	MED Events	MED Total	Weight	Risk Ratio M-H, Fixed, 95% CI
**13.2.1 manual acupuncture**						
Chen 2011	58	62	50	66	7.0%	1.23 [1.06, 1.44]
Chen 2012	48	54	42	58	5.9%	1.23 [1.02, 1.48]
Chen 2019	48	50	42	50	6.1%	1.14 [1.00, 1.31]
Fan 2015	50	56	34	56	4.9%	1.47 [1.17, 1.85]
Gao 2016	48	50	39	50	5.7%	1.23 [1.05, 1.44]
He 2012	126	130	102	130	14.8%	1.24 [1.12, 1.36]
Jiang 2017	42	45	35	45	5.1%	1.20 [1.01, 1.43]
Liu 2011	37	40	30	38	4.5%	1.17 [0.97, 1.41]
Wang 2019	20	21	14	21	2.0%	1.43 [1.04, 1.96]
Yan 2016	64	68	55	68	8.0%	1.16 [1.02, 1.33]
Yu 2008	35	36	25	34	3.7%	1.32 [1.07, 1.63]
Zhang 2010	28	31	24	30	3.5%	1.13 [0.91, 1.40]
Zhang 2013	31	36	22	36	3.2%	1.41 [1.05, 1.89]
Zhang 2014a	27	30	23	28	3.5%	1.10 [0.89, 1.35]
Zhang 2014b	37	40	26	36	4.0%	1.28 [1.03, 1.60]
**Subtotal (95% CI)**		**749**		**746**	**81.9%**	**1.24 [1.18, 1.29]**
Total events	699		563			
Heterogeneity: Chi² = 8.84, df = 14 (P = 0.84); I² = 0%						
Test for overall effect: Z = 9.25 (P < 0.00001)						
**13.2.2 electroacupuncture**						
Chung 2019	39	66	11	66	1.6%	3.55 [1.99, 6.30]
Han 2020(part1)	31	39	27	40	3.9%	1.18 [0.90, 1.54]
Han 2020(part2)	42	43	32	41	4.8%	1.25 [1.06, 1.48]
Mao 2014	35	40	21	40	3.1%	1.67 [1.21, 2.29]
**Subtotal (95% CI)**		**188**		**187**	**13.3%**	**1.60 [1.37, 1.87]**
Total events	147		91			
Heterogeneity: Chi² = 20.65, df = 3 (P = 0.0001); I² = 85%						
Test for overall effect: Z = 6.02 (P < 0.00001)						
**13.2.3 auricular acupuncture**						
Chen 2017	41	45	33	45	4.8%	1.24 [1.02, 1.52]
**Subtotal (95% CI)**		**45**		**45**	**4.8%**	**1.24 [1.02, 1.52]**
Total events	41		33			
Heterogeneity: Not applicable						
Test for overall effect: Z = 2.14 (P = 0.03)						
**Total (95% CI)**		**982**		**978**	**100.0%**	**1.29 [1.23, 1.34]**
Total events	887		687			
Heterogeneity: Chi² = 29.18, df = 19 (P = 0.06); I² = 35%						
Test for overall effect: Z = 11.25 (P < 0.00001)						
Test for subgroup differences: Chi² = 10.10, df = 2 (P = 0.006), I² = 80.2%						

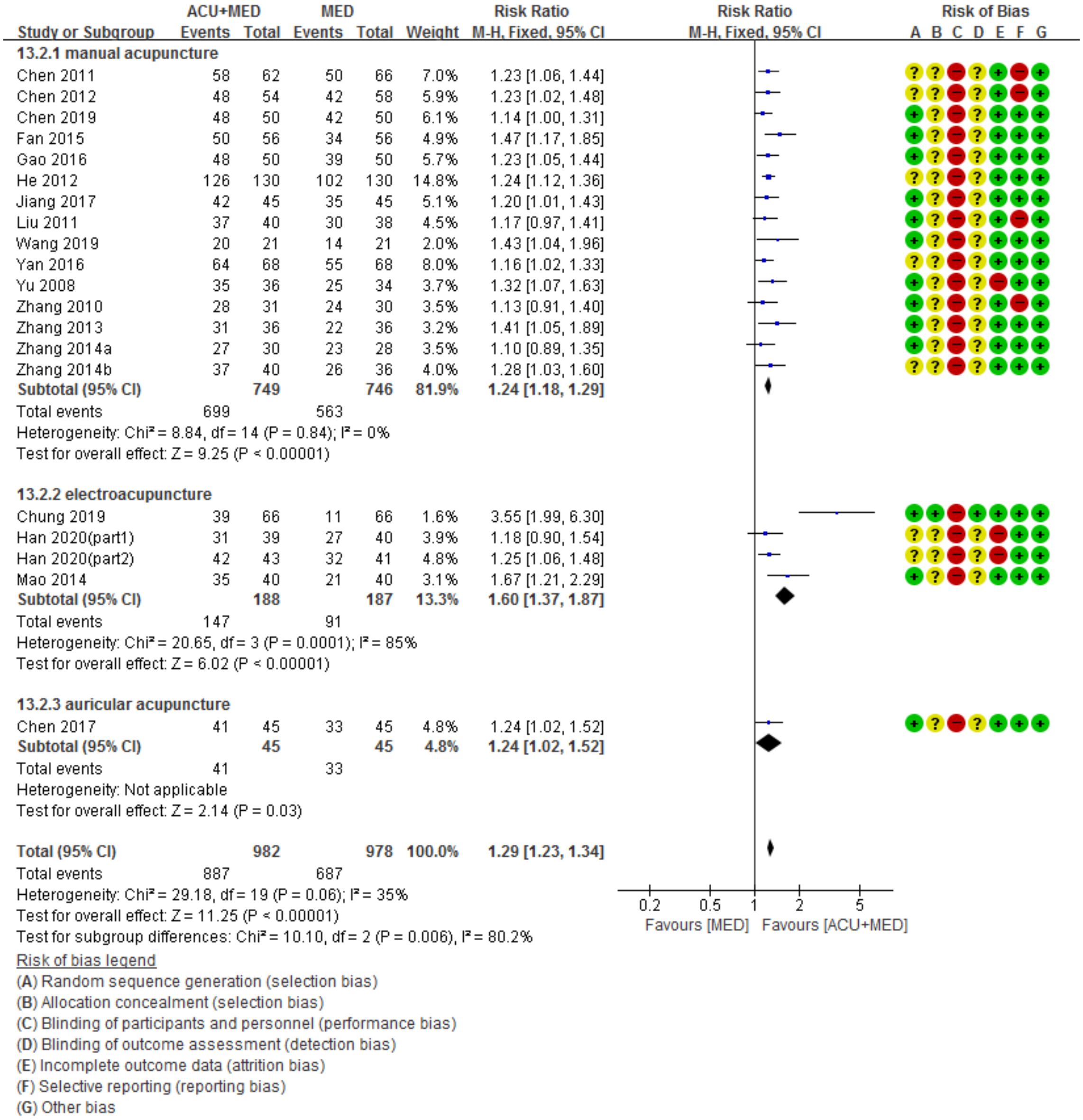

Risk of bias legend
(A) Random sequence generation (selection bias)
(B) Allocation concealment (selection bias)
(C) Blinding of participants and personnel (performance bias)
(D) Blinding of outcome assessment (detection bias)
(E) Incomplete outcome data (attrition bias)
(F) Selective reporting (reporting bias)
(G) Other bias

3) SF-36 total score (subgroup analysis according to WM type)

Study or Subgroup	ACU+MED Mean	ACU+MED SD	ACU+MED Total	MED Mean	MED SD	MED Total	Weight	Mean Difference IV, Fixed, 95% CI
**12.3.1 prokinetics**								
Jiang 2017	85.75	3.96	45	79.81	4.37	45	83.7%	5.94 [4.22, 7.66]
**Subtotal (95% CI)**			**45**			**45**	**83.7%**	**5.94 [4.22, 7.66]**
Heterogeneity: Not applicable								
Test for overall effect: Z = 6.76 (P < 0.00001)								
**12.3.2 prokinetics&acid suppressants**								
Gao 2016	93.25	10.34	50	81.47	9.57	50	16.3%	11.78 [7.87, 15.69]
**Subtotal (95% CI)**			**50**			**50**	**16.3%**	**11.78 [7.87, 15.69]**
Heterogeneity: Not applicable								
Test for overall effect: Z = 5.91 (P < 0.00001)								
**Total (95% CI)**			**95**			**95**	**100.0%**	**6.89 [5.32, 8.47]**

Heterogeneity: Chi² = 7.19, df = 1 (P = 0.007); I² = 86%
Test for overall effect: Z = 8.57 (P < 0.00001)
Test for subgroup differences: Chi² = 7.19, df = 1 (P = 0.007), I² = 86.1%

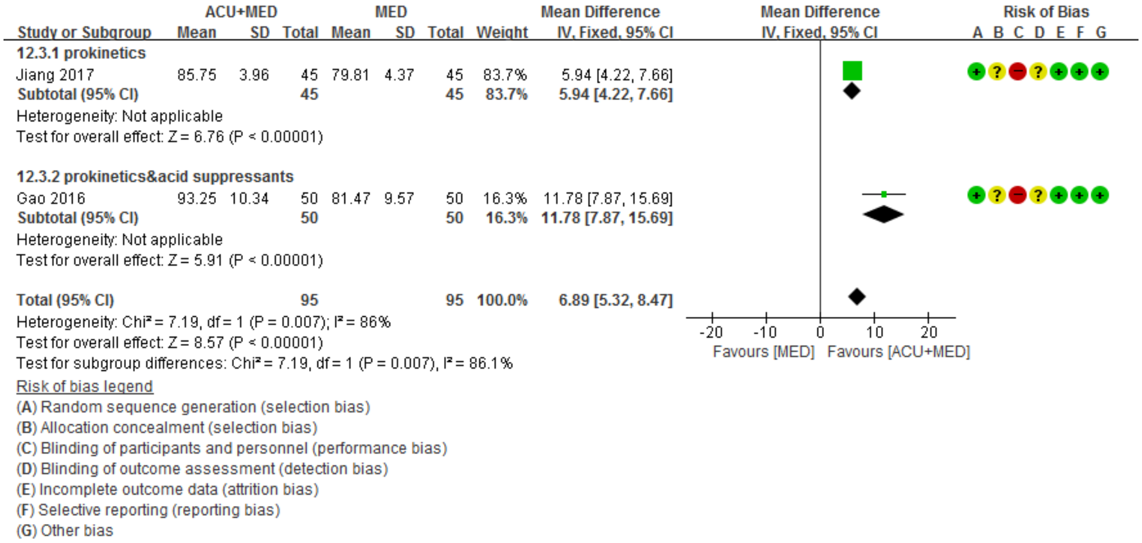

Risk of bias legend
(A) Random sequence generation (selection bias)
(B) Allocation concealment (selection bias)
(C) Blinding of participants and personnel (performance bias)
(D) Blinding of outcome assessment (detection bias)
(E) Incomplete outcome data (attrition bias)
(F) Selective reporting (reporting bias)
(G) Other bias

4) SF-36 vitality (subgroup analysis according to WM type)

Study or Subgroup	ACU+MED Mean	ACU+MED SD	ACU+MED Total	MED Mean	MED SD	MED Total	Weight	Mean Difference IV, Fixed, 95% CI
**12.4.1 prokinetics**								
Mao 2014	77.75	9.2	40	69.25	11.24	40	22.9%	8.50 [4.00, 13.00]
**Subtotal (95% CI)**			**40**			**40**	**22.9%**	**8.50 [4.00, 13.00]**
Heterogeneity: Not applicable								
Test for overall effect: Z = 3.70 (P = 0.0002)								
**12.4.2 prokinetics&antidepressants**								
Yang 2017	54.2	6.5	50	50.6	6	50	77.1%	3.60 [1.15, 6.05]
**Subtotal (95% CI)**			**50**			**50**	**77.1%**	**3.60 [1.15, 6.05]**
Heterogeneity: Not applicable								
Test for overall effect: Z = 2.88 (P = 0.004)								
**Total (95% CI)**			**90**			**90**	**100.0%**	**4.72 [2.57, 6.87]**

Heterogeneity: Chi² = 3.51, df = 1 (P = 0.06); I² = 72%
Test for overall effect: Z = 4.30 (P < 0.0001)
Test for subgroup differences: Chi² = 3.51, df = 1 (P = 0.06), I² = 71.5%

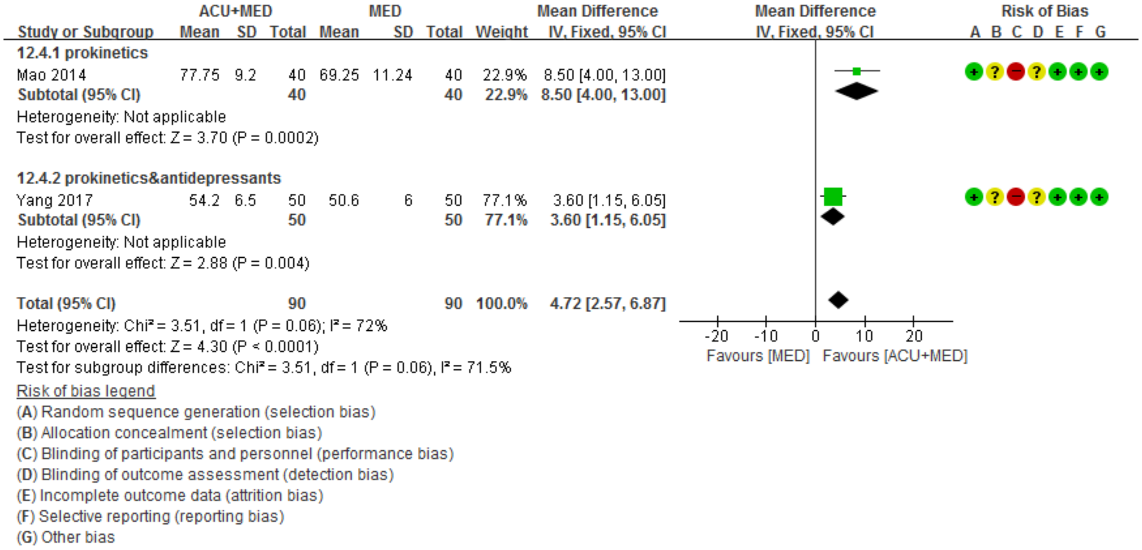

Risk of bias legend
(A) Random sequence generation (selection bias)
(B) Allocation concealment (selection bias)
(C) Blinding of participants and personnel (performance bias)
(D) Blinding of outcome assessment (detection bias)
(E) Incomplete outcome data (attrition bias)
(F) Selective reporting (reporting bias)
(G) Other bias

5) SF-36 physical functioning (subgroup analysis according to WM type)

Study or Subgroup	ACU+MED Mean	ACU+MED SD	ACU+MED Total	MED Mean	MED SD	MED Total	Weight	Mean Difference IV, Fixed, 95% CI
**12.5.1 prokinetics**								
Mao 2014	85.63	10.51	40	80.25	11.93	40	37.1%	5.38 [0.45, 10.31]
**Subtotal (95% CI)**			**40**			**40**	**37.1%**	**5.38 [0.45, 10.31]**
Heterogeneity: Not applicable								
Test for overall effect: Z = 2.14 (P = 0.03)								
**12.5.2 prokinetics&antidepressants**								
Yang 2017	87.9	9.8	50	83.7	9.5	50	62.9%	4.20 [0.42, 7.98]
**Subtotal (95% CI)**			**50**			**50**	**62.9%**	**4.20 [0.42, 7.98]**
Heterogeneity: Not applicable								
Test for overall effect: Z = 2.18 (P = 0.03)								
**Total (95% CI)**			**90**			**90**	**100.0%**	**4.64 [1.64, 7.64]**

Heterogeneity: Chi² = 0.14, df = 1 (P = 0.71); I² = 0%
Test for overall effect: Z = 3.03 (P = 0.002)
Test for subgroup differences: Chi² = 0.14, df = 1 (P = 0.71), I² = 0%

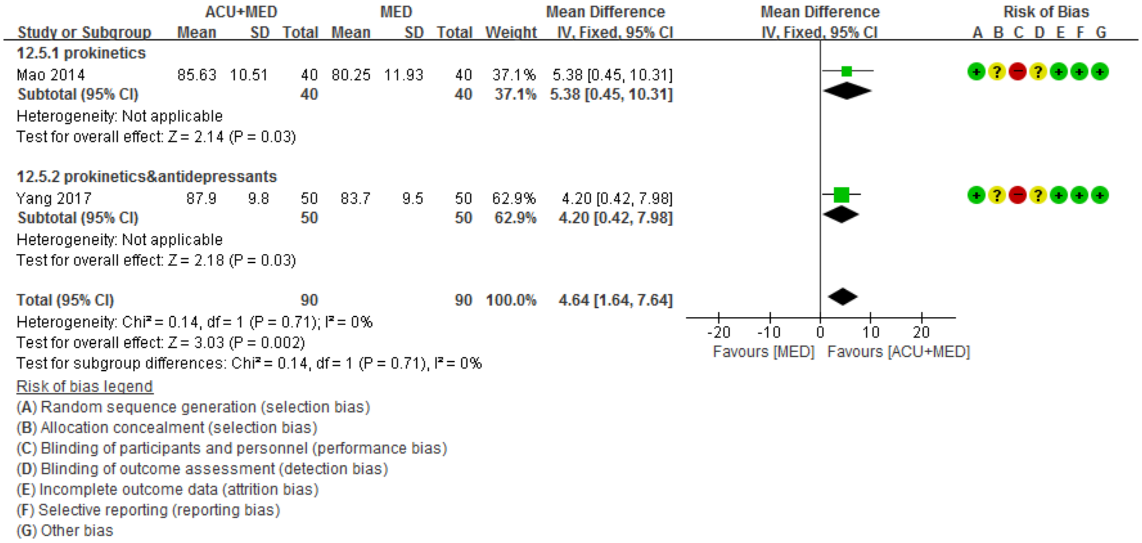

Risk of bias legend
(A) Random sequence generation (selection bias)
(B) Allocation concealment (selection bias)
(C) Blinding of participants and personnel (performance bias)
(D) Blinding of outcome assessment (detection bias)
(E) Incomplete outcome data (attrition bias)
(F) Selective reporting (reporting bias)
(G) Other bias

6) SF-36 bodily pain (subgroup analysis according to WM type)

Study or Subgroup	ACU+MED Mean	ACU+MED SD	ACU+MED Total	MED Mean	MED SD	MED Total	Weight	Mean Difference IV, Fixed, 95% CI
**12.6.1 prokinetics**								
Mao 2014	80.63	13.8	40	75.23	9.17	40	22.7%	5.40 [0.27, 10.53]
**Subtotal (95% CI)**			**40**			**40**	**22.7%**	**5.40 [0.27, 10.53]**
Heterogeneity: Not applicable								
Test for overall effect: Z = 2.06 (P = 0.04)								
**12.6.2 prokinetics&antidepressants**								
Yang 2017	61.5	7.2	50	59.4	7	50	77.3%	2.10 [-0.68, 4.88]
**Subtotal (95% CI)**			**50**			**50**	**77.3%**	**2.10 [-0.68, 4.88]**
Heterogeneity: Not applicable								
Test for overall effect: Z = 1.48 (P = 0.14)								
**Total (95% CI)**			**90**			**90**	**100.0%**	**2.85 [0.40, 5.30]**

Heterogeneity: Chi² = 1.23, df = 1 (P = 0.27); I² = 18%
Test for overall effect: Z = 2.28 (P = 0.02)
Test for subgroup differences: Chi² = 1.23, df = 1 (P = 0.27), I² = 18.5%

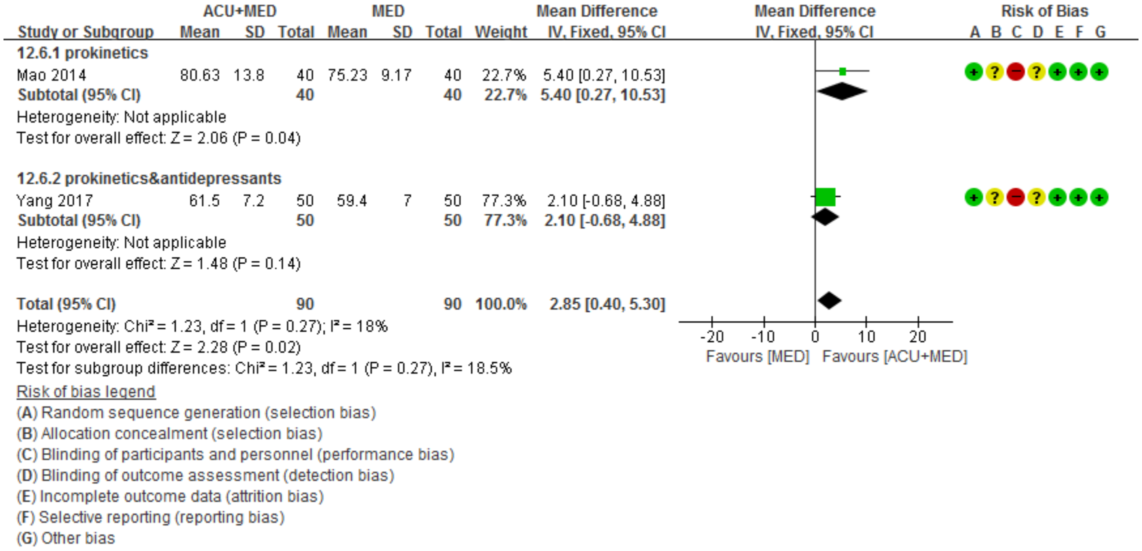

Risk of bias legend
(A) Random sequence generation (selection bias)
(B) Allocation concealment (selection bias)
(C) Blinding of participants and personnel (performance bias)
(D) Blinding of outcome assessment (detection bias)
(E) Incomplete outcome data (attrition bias)
(F) Selective reporting (reporting bias)
(G) Other bias

7) SF-36 general health perceptions (subgroup analysis according to WM type)

Study or Subgroup	ACU+MED Mean	ACU+MED SD	ACU+MED Total	MED Mean	MED SD	MED Total	Weight	Mean Difference IV, Fixed, 95% CI
**12.7.1 prokinetics**								
Mao 2014	79	10.51	40	79.88	12.06	40	21.4%	-0.88 [-5.84, 4.08]
**Subtotal (95% CI)**			**40**			**40**	**21.4%**	**-0.88 [-5.84, 4.08]**
Heterogeneity: Not applicable								
Test for overall effect: Z = 0.35 (P = 0.73)								
**12.7.2 prokinetics&antidepressants**								
Yang 2017	58.2	6.8	50	53.2	6.4	50	78.6%	5.00 [2.41, 7.59]
**Subtotal (95% CI)**			**50**			**50**	**78.6%**	**5.00 [2.41, 7.59]**
Heterogeneity: Not applicable								
Test for overall effect: Z = 3.79 (P = 0.0002)								
**Total (95% CI)**			**90**			**90**	**100.0%**	**3.74 [1.45, 6.03]**

Heterogeneity: Chi² = 4.25, df = 1 (P = 0.04); I² = 76%
Test for overall effect: Z = 3.20 (P = 0.001)
Test for subgroup differences: Chi² = 4.25, df = 1 (P = 0.04), I² = 76.5%

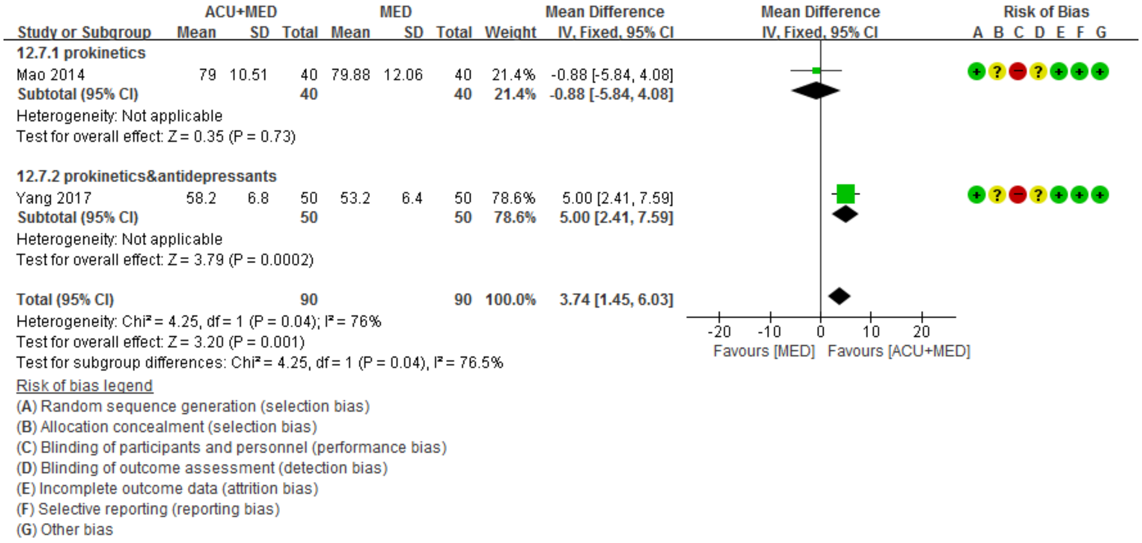

Risk of bias legend
(A) Random sequence generation (selection bias)
(B) Allocation concealment (selection bias)
(C) Blinding of participants and personnel (performance bias)
(D) Blinding of outcome assessment (detection bias)
(E) Incomplete outcome data (attrition bias)
(F) Selective reporting (reporting bias)
(G) Other bias

8) SF-36 physical role functioning (subgroup analysis according to WM type)

Study or Subgroup	ACU+MED Mean	ACU+MED SD	ACU+MED Total	MED Mean	MED SD	MED Total	Weight	Mean Difference IV, Fixed, 95% CI
**12.8.1 prokinetics**								
Mao 2014	78.13	15.18	40	68.75	20.22	40	9.3%	9.38 [1.54, 17.22]
**Subtotal (95% CI)**			**40**			**40**	**9.3%**	**9.38 [1.54, 17.22]**
Heterogeneity: Not applicable								
Test for overall effect: Z = 2.35 (P = 0.02)								
**12.8.2 prokinetics&antidepressants**								
Yang 2017	55.2	6.5	50	52.6	6.3	50	90.7%	2.60 [0.09, 5.11]
**Subtotal (95% CI)**			**50**			**50**	**90.7%**	**2.60 [0.09, 5.11]**
Heterogeneity: Not applicable								
Test for overall effect: Z = 2.03 (P = 0.04)								
**Total (95% CI)**			**90**			**90**	**100.0%**	**3.23 [0.84, 5.62]**

Heterogeneity: Chi² = 2.61, df = 1 (P = 0.11); I² = 62%
Test for overall effect: Z = 2.65 (P = 0.008)
Test for subgroup differences: Chi² = 2.61, df = 1 (P = 0.11), I² = 61.7%

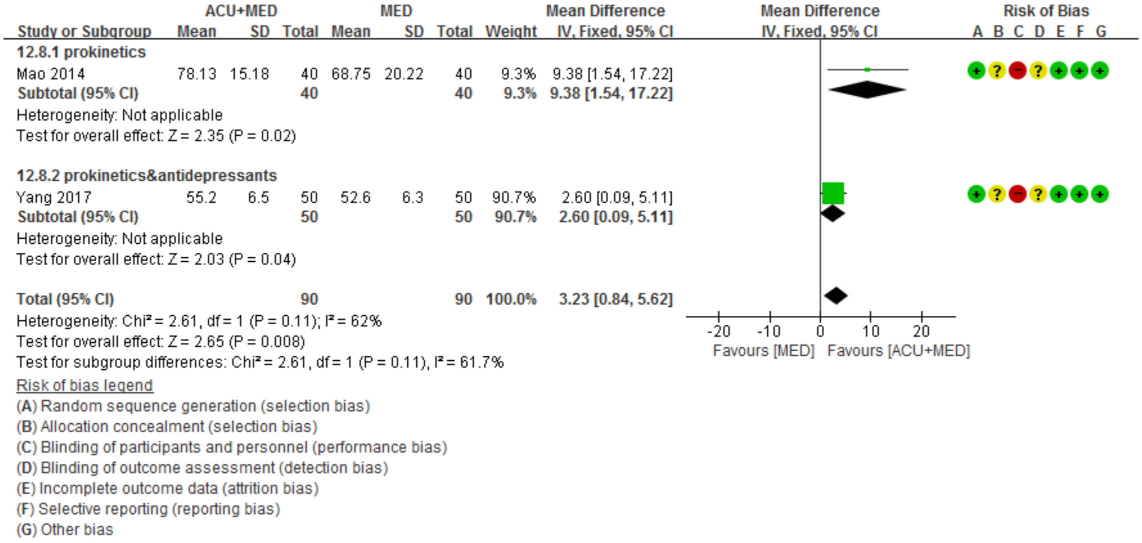

Risk of bias legend
(A) Random sequence generation (selection bias)
(B) Allocation concealment (selection bias)
(C) Blinding of participants and personnel (performance bias)
(D) Blinding of outcome assessment (detection bias)
(E) Incomplete outcome data (attrition bias)
(F) Selective reporting (reporting bias)
(G) Other bias

9) SF-36 emotional role functioning (subgroup analysis according to WM type)

Study or Subgroup	ACU+MED Mean	ACU+MED SD	ACU+MED Total	MED Mean	MED SD	MED Total	Weight	Mean Difference IV, Fixed, 95% CI	Risk of Bias A B C D E F G
**12.9.1 prokinetics**									
Mao 2014	68.3	26.11	40	50.8	29.22	40	4.3%	17.50 [5.36, 29.64]	+ ? − ? + + +
**Subtotal (95% CI)**			**40**			**40**	**4.3%**	**17.50 [5.36, 29.64]**	
Heterogeneity: Not applicable									
Test for overall effect: Z = 2.82 (P = 0.005)									
**12.9.2 prokinetics&antidepressants**									
Yang 2017	56.8	6.7	50	54.1	6.5	50	95.7%	2.70 [0.11, 5.29]	+ ? − ? + + +
**Subtotal (95% CI)**			**50**			**50**	**95.7%**	**2.70 [0.11, 5.29]**	
Heterogeneity: Not applicable									
Test for overall effect: Z = 2.05 (P = 0.04)									
**Total (95% CI)**			**90**			**90**	**100.0%**	**3.34 [0.81, 5.87]**	

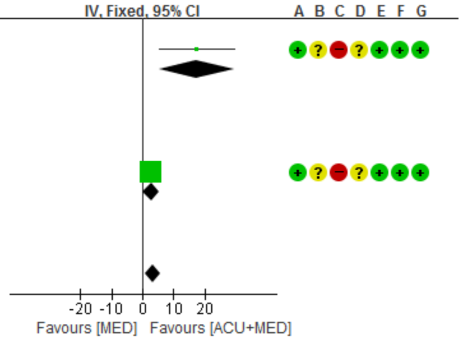

Heterogeneity: Chi² = 5.46, df = 1 (P = 0.02); I² = 82%
Test for overall effect: Z = 2.59 (P = 0.010)
Test for subgroup differences: Chi² = 5.46, df = 1 (P = 0.02), I² = 81.7%

Risk of bias legend
(A) Random sequence generation (selection bias)
(B) Allocation concealment (selection bias)
(C) Blinding of participants and personnel (performance bias)
(D) Blinding of outcome assessment (detection bias)
(E) Incomplete outcome data (attrition bias)
(F) Selective reporting (reporting bias)
(G) Other bias

10) SF-36 social role functioning (subgroup analysis according to WM type)

Study or Subgroup	ACU+MED Mean	ACU+MED SD	ACU+MED Total	MED Mean	MED SD	MED Total	Weight	Mean Difference IV, Fixed, 95% CI	Risk of Bias A B C D E F G
**12.10.1 prokinetics**									
Mao 2014	77.81	15.63	40	70.94	13.98	40	15.1%	6.87 [0.37, 13.37]	+ ? − ? + + +
**Subtotal (95% CI)**			**40**			**40**	**15.1%**	**6.87 [0.37, 13.37]**	
Heterogeneity: Not applicable									
Test for overall effect: Z = 2.07 (P = 0.04)									
**12.10.2 prokinetics&antidepressants**									
Yang 2017	60	7.1	50	58.5	6.9	50	84.9%	1.50 [-1.24, 4.24]	+ ? − ? + + +
**Subtotal (95% CI)**			**50**			**50**	**84.9%**	**1.50 [-1.24, 4.24]**	
Heterogeneity: Not applicable									
Test for overall effect: Z = 1.07 (P = 0.28)									
**Total (95% CI)**			**90**			**90**	**100.0%**	**2.31 [-0.22, 4.84]**	

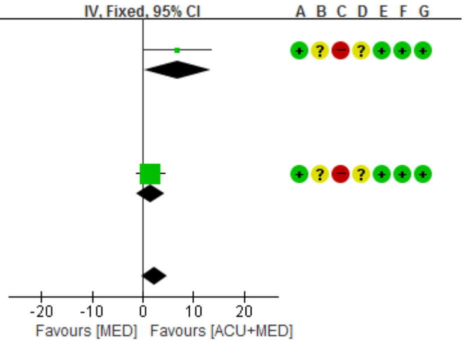

Heterogeneity: Chi² = 2.23, df = 1 (P = 0.14); I² = 55%
Test for overall effect: Z = 1.79 (P = 0.07)
Test for subgroup differences: Chi² = 2.23, df = 1 (P = 0.14), I² = 55.1%

Risk of bias legend
(A) Random sequence generation (selection bias)
(B) Allocation concealment (selection bias)
(C) Blinding of participants and personnel (performance bias)
(D) Blinding of outcome assessment (detection bias)
(E) Incomplete outcome data (attrition bias)
(F) Selective reporting (reporting bias)
(G) Other bias

11) SF-36 mental health (subgroup analysis according to WM type)

Study or Subgroup	ACU+MED Mean	ACU+MED SD	ACU+MED Total	MED Mean	MED SD	MED Total	Weight	Mean Difference IV, Fixed, 95% CI	Risk of Bias A B C D E F G
**12.11.1 prokinetics**									
Mao 2014	62.6	13.93	40	51.6	13.28	40	17.5%	11.00 [5.04, 16.96]	+ ? − ? + + +
**Subtotal (95% CI)**			**40**			**40**	**17.5%**	**11.00 [5.04, 16.96]**	
Heterogeneity: Not applicable									
Test for overall effect: Z = 3.61 (P = 0.0003)									
**12.11.2 prokinetics&antidepressants**									
Yang 2017	63.6	7.4	50	55.8	6.6	50	82.5%	7.80 [5.05, 10.55]	+ ? − ? + + +
**Subtotal (95% CI)**			**50**			**50**	**82.5%**	**7.80 [5.05, 10.55]**	
Heterogeneity: Not applicable									
Test for overall effect: Z = 5.56 (P < 0.00001)									
**Total (95% CI)**			**90**			**90**	**100.0%**	**8.36 [5.86, 10.86]**	

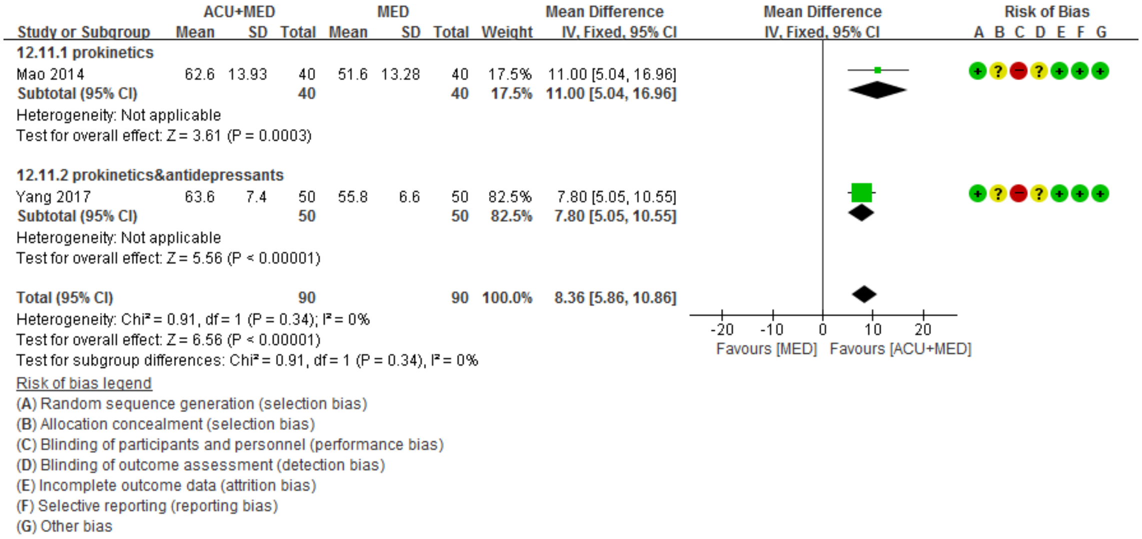

Heterogeneity: Chi² = 0.91, df = 1 (P = 0.34); I² = 0%
Test for overall effect: Z = 6.56 (P < 0.00001)
Test for subgroup differences: Chi² = 0.91, df = 1 (P = 0.34), I² = 0%

Risk of bias legend
(A) Random sequence generation (selection bias)
(B) Allocation concealment (selection bias)
(C) Blinding of participants and personnel (performance bias)
(D) Blinding of outcome assessment (detection bias)
(E) Incomplete outcome data (attrition bias)
(F) Selective reporting (reporting bias)
(G) Other bias

12) SF-36 total score (subgroup analysis according to acupuncture type)

Study or Subgroup	ACU+MED Mean	ACU+MED SD	ACU+MED Total	MED Mean	MED SD	MED Total	Weight	Mean Difference IV, Fixed, 95% CI	Risk of Bias A B C D E F G
**13.3.1 manual acupuncture**									
Gao 2016	93.25	10.34	50	81.47	9.57	50	16.3%	11.78 [7.87, 15.69]	+ ? − ? + + +
Jiang 2017	85.75	3.96	45	79.81	4.37	45	83.7%	5.94 [4.22, 7.66]	+ ? − ? + + +
**Subtotal (95% CI)**			**95**			**95**	**100.0%**	**6.89 [5.32, 8.47]**	
Heterogeneity: Chi² = 7.19, df = 1 (P = 0.007); I² = 86%									
Test for overall effect: Z = 8.57 (P < 0.00001)									
**Total (95% CI)**			**95**			**95**	**100.0%**	**6.89 [5.32, 8.47]**	

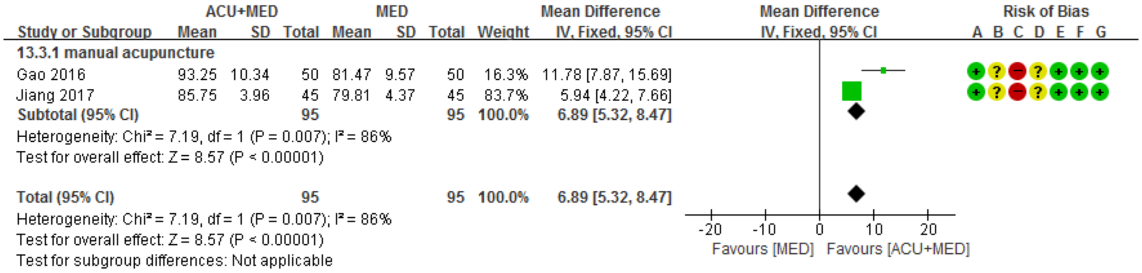

Heterogeneity: Chi² = 7.19, df = 1 (P = 0.007); I² = 86%
Test for overall effect: Z = 8.57 (P < 0.00001)
Test for subgroup differences: Not applicable

Risk of bias legend
(A) Random sequence generation (selection bias)
(B) Allocation concealment (selection bias)
(C) Blinding of participants and personnel (performance bias)
(D) Blinding of outcome assessment (detection bias)
(E) Incomplete outcome data (attrition bias)
(F) Selective reporting (reporting bias)
(G) Other bias

13) SF-36 vitality (subgroup analysis according to acupuncture type)

Study or Subgroup	ACU+MED Mean	ACU+MED SD	ACU+MED Total	MED Mean	MED SD	MED Total	Weight	Mean Difference IV, Fixed, 95% CI
**13.4.1 manual acupuncture**								
Yang 2017	54.2	6.5	50	50.6	6	50	77.1%	3.60 [1.15, 6.05]
**Subtotal (95% CI)**			**50**			**50**	**77.1%**	**3.60 [1.15, 6.05]**
Heterogeneity: Not applicable								
Test for overall effect: Z = 2.88 (P = 0.004)								
**13.4.2 electroacupuncture**								
Mao 2014	77.75	9.2	40	69.25	11.24	40	22.9%	8.50 [4.00, 13.00]
**Subtotal (95% CI)**			**40**			**40**	**22.9%**	**8.50 [4.00, 13.00]**
Heterogeneity: Not applicable								
Test for overall effect: Z = 3.70 (P = 0.0002)								
**Total (95% CI)**			**90**			**90**	**100.0%**	**4.72 [2.57, 6.87]**

Heterogeneity: Chi² = 3.51, df = 1 (P = 0.06); I² = 72%
Test for overall effect: Z = 4.30 (P < 0.0001)
Test for subgroup differences: Chi² = 3.51, df = 1 (P = 0.06), I² = 71.5%

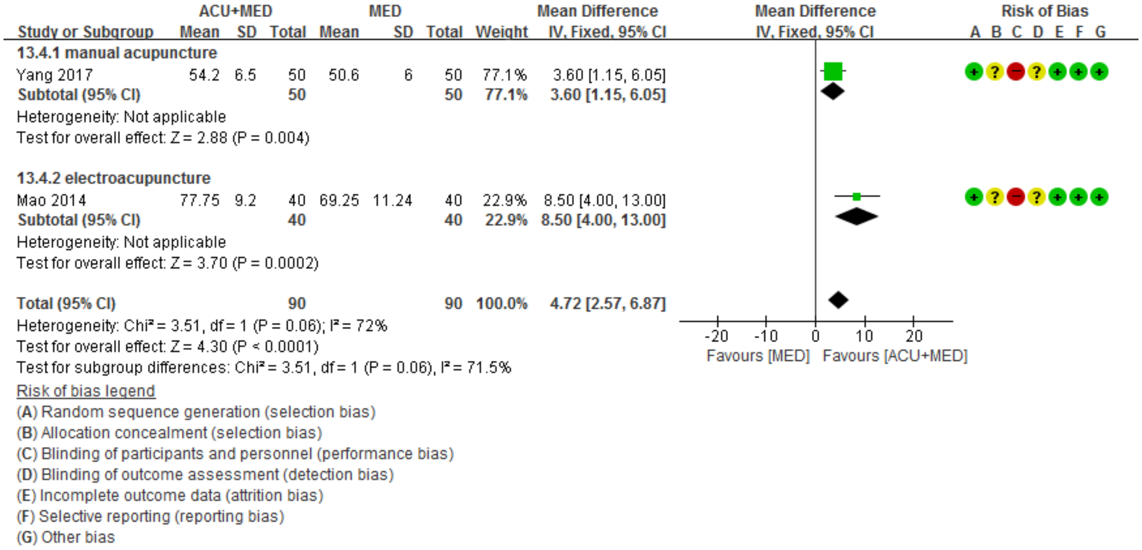

Risk of bias legend
(A) Random sequence generation (selection bias)
(B) Allocation concealment (selection bias)
(C) Blinding of participants and personnel (performance bias)
(D) Blinding of outcome assessment (detection bias)
(E) Incomplete outcome data (attrition bias)
(F) Selective reporting (reporting bias)
(G) Other bias

14) SF-36 physical functioning (subgroup analysis according to acupuncture type)

Study or Subgroup	ACU+MED Mean	ACU+MED SD	ACU+MED Total	MED Mean	MED SD	MED Total	Weight	Mean Difference IV, Fixed, 95% CI
**13.5.1 manual acupuncture**								
Yang 2017	87.9	9.8	50	83.7	9.5	50	62.9%	4.20 [0.42, 7.98]
**Subtotal (95% CI)**			**50**			**50**	**62.9%**	**4.20 [0.42, 7.98]**
Heterogeneity: Not applicable								
Test for overall effect: Z = 2.18 (P = 0.03)								
**13.5.2 electroacupuncture**								
Mao 2014	85.63	10.51	40	80.25	11.93	40	37.1%	5.38 [0.45, 10.31]
**Subtotal (95% CI)**			**40**			**40**	**37.1%**	**5.38 [0.45, 10.31]**
Heterogeneity: Not applicable								
Test for overall effect: Z = 2.14 (P = 0.03)								
**Total (95% CI)**			**90**			**90**	**100.0%**	**4.64 [1.64, 7.64]**

Heterogeneity: Chi² = 0.14, df = 1 (P = 0.71); I² = 0%
Test for overall effect: Z = 3.03 (P = 0.002)
Test for subgroup differences: Chi² = 0.14, df = 1 (P = 0.71), I² = 0%

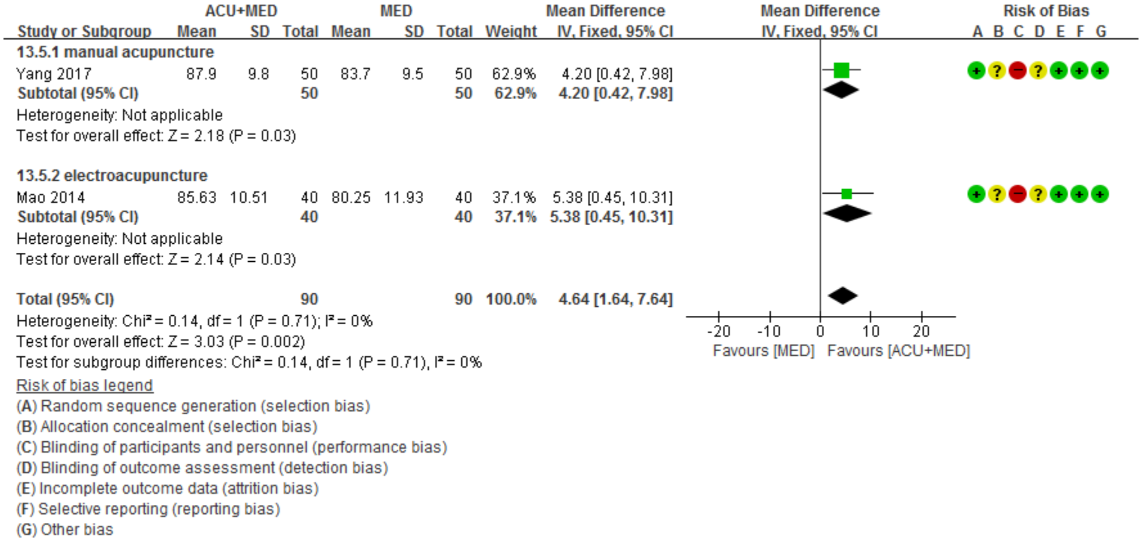

Risk of bias legend
(A) Random sequence generation (selection bias)
(B) Allocation concealment (selection bias)
(C) Blinding of participants and personnel (performance bias)
(D) Blinding of outcome assessment (detection bias)
(E) Incomplete outcome data (attrition bias)
(F) Selective reporting (reporting bias)
(G) Other bias

15) SF-36 bodily pain (subgroup analysis according to acupuncture type)

Study or Subgroup	ACU+MED Mean	ACU+MED SD	ACU+MED Total	MED Mean	MED SD	MED Total	Weight	Mean Difference IV, Fixed, 95% CI
**13.6.1 manual acupuncture**								
Yang 2017	61.5	7.2	50	59.4	7	50	77.3%	2.10 [-0.68, 4.88]
**Subtotal (95% CI)**			**50**			**50**	**77.3%**	**2.10 [-0.68, 4.88]**
Heterogeneity: Not applicable								
Test for overall effect: Z = 1.48 (P = 0.14)								
**13.6.2 electroacupuncture**								
Mao 2014	80.63	13.8	40	75.23	9.17	40	22.7%	5.40 [0.27, 10.53]
**Subtotal (95% CI)**			**40**			**40**	**22.7%**	**5.40 [0.27, 10.53]**
Heterogeneity: Not applicable								
Test for overall effect: Z = 2.06 (P = 0.04)								
**Total (95% CI)**			**90**			**90**	**100.0%**	**2.85 [0.40, 5.30]**

Heterogeneity: Chi² = 1.23, df = 1 (P = 0.27); I² = 18%
Test for overall effect: Z = 2.28 (P = 0.02)
Test for subgroup differences: Chi² = 1.23, df = 1 (P = 0.27), I² = 18.5%

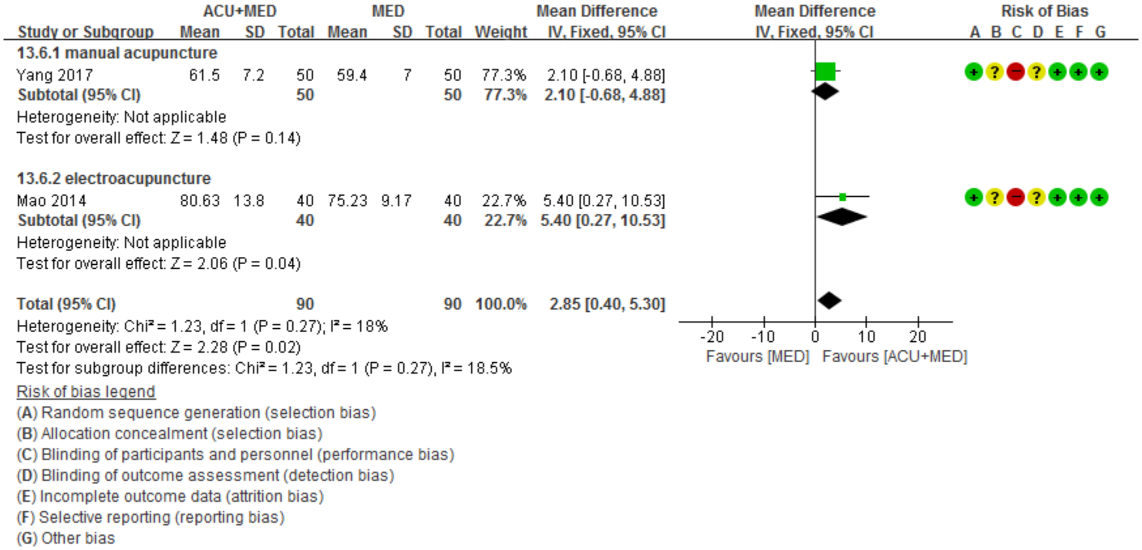

Risk of bias legend
(A) Random sequence generation (selection bias)
(B) Allocation concealment (selection bias)
(C) Blinding of participants and personnel (performance bias)
(D) Blinding of outcome assessment (detection bias)
(E) Incomplete outcome data (attrition bias)
(F) Selective reporting (reporting bias)
(G) Other bias

16) SF-36 general health perceptions (subgroup analysis according to acupuncture type)

Study or Subgroup	ACU+MED Mean	ACU+MED SD	ACU+MED Total	MED Mean	MED SD	MED Total	Weight	Mean Difference IV, Fixed, 95% CI
**13.7.1 manual acupuncture**								
Yang 2017	58.2	6.8	50	53.2	6.4	50	78.6%	5.00 [2.41, 7.59]
**Subtotal (95% CI)**			**50**			**50**	**78.6%**	**5.00 [2.41, 7.59]**
Heterogeneity: Not applicable								
Test for overall effect: Z = 3.79 (P = 0.0002)								
**13.7.2 electroacupuncture**								
Mao 2014	79	10.51	40	79.88	12.06	40	21.4%	-0.88 [-5.84, 4.08]
**Subtotal (95% CI)**			**40**			**40**	**21.4%**	**-0.88 [-5.84, 4.08]**
Heterogeneity: Not applicable								
Test for overall effect: Z = 0.35 (P = 0.73)								
**Total (95% CI)**			**90**			**90**	**100.0%**	**3.74 [1.45, 6.03]**

Heterogeneity: Chi² = 4.25, df = 1 (P = 0.04); I² = 76%
Test for overall effect: Z = 3.20 (P = 0.001)
Test for subgroup differences: Chi² = 4.25, df = 1 (P = 0.04), I² = 76.5%

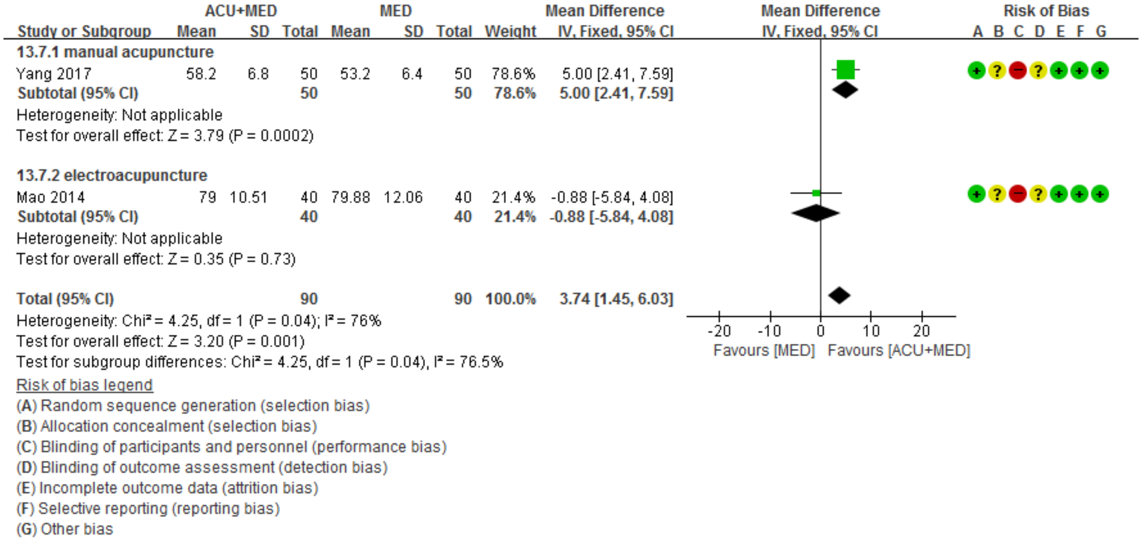

Risk of bias legend
(A) Random sequence generation (selection bias)
(B) Allocation concealment (selection bias)
(C) Blinding of participants and personnel (performance bias)
(D) Blinding of outcome assessment (detection bias)
(E) Incomplete outcome data (attrition bias)
(F) Selective reporting (reporting bias)
(G) Other bias

17) SF-36 physical role functioning (subgroup analysis according to acupuncture type)

	ACU+MED			MED				Mean Difference
Study or Subgroup	Mean	SD	Total	Mean	SD	Total	Weight	IV, Fixed, 95% CI
**13.8.1 manual acupuncture**								
Yang 2017	55.2	6.5	50	52.6	6.3	50	90.7%	2.60 [0.09, 5.11]
**Subtotal (95% CI)**			**50**			**50**	**90.7%**	**2.60 [0.09, 5.11]**
Heterogeneity: Not applicable								
Test for overall effect: Z = 2.03 (P = 0.04)								
**13.8.2 electroacupuncture**								
Mao 2014	78.13	15.18	40	68.75	20.22	40	9.3%	9.38 [1.54, 17.22]
**Subtotal (95% CI)**			**40**			**40**	**9.3%**	**9.38 [1.54, 17.22]**
Heterogeneity: Not applicable								
Test for overall effect: Z = 2.35 (P = 0.02)								
**Total (95% CI)**			**90**			**90**	**100.0%**	**3.23 [0.84, 5.62]**
Heterogeneity: $Chi^2 = 2.61$, df = 1 (P = 0.11); $I^2 = 62\%$								
Test for overall effect: Z = 2.65 (P = 0.008)								
Test for subgroup differences: $Chi^2 = 2.61$, df = 1 (P = 0.11), $I^2 = 61.7\%$								

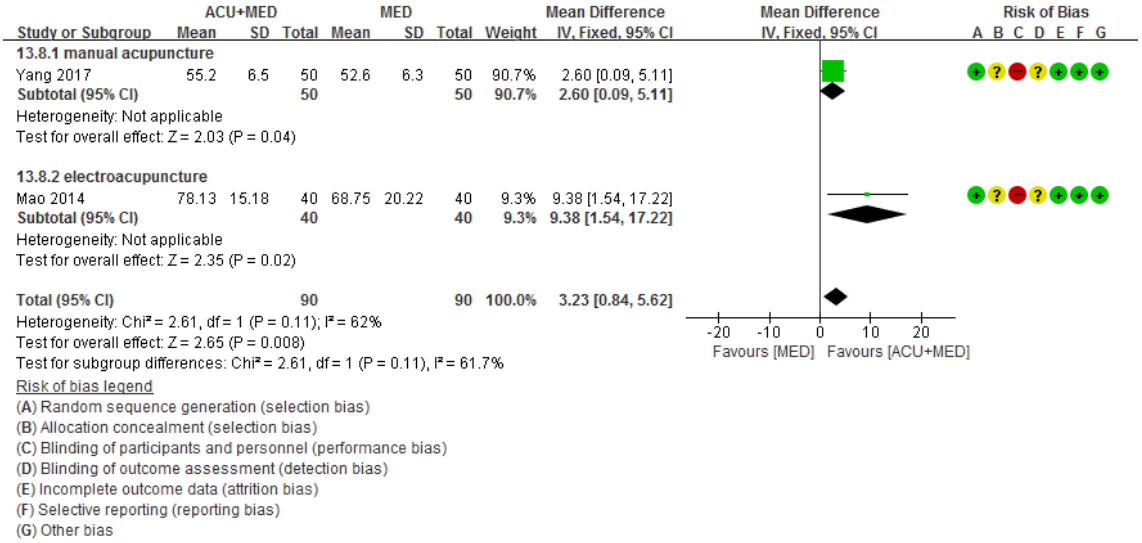

Risk of bias legend
(A) Random sequence generation (selection bias)
(B) Allocation concealment (selection bias)
(C) Blinding of participants and personnel (performance bias)
(D) Blinding of outcome assessment (detection bias)
(E) Incomplete outcome data (attrition bias)
(F) Selective reporting (reporting bias)
(G) Other bias

18) SF-36 emotional role functioning (subgroup analysis according to acupuncture type)

	ACU+MED			MED				Mean Difference
Study or Subgroup	Mean	SD	Total	Mean	SD	Total	Weight	IV, Fixed, 95% CI
**13.9.1 manual acupuncture**								
Yang 2017	56.8	6.7	50	54.1	6.5	50	95.7%	2.70 [0.11, 5.29]
**Subtotal (95% CI)**			**50**			**50**	**95.7%**	**2.70 [0.11, 5.29]**
Heterogeneity: Not applicable								
Test for overall effect: Z = 2.05 (P = 0.04)								
**13.9.2 electroacupuncture**								
Mao 2014	68.3	26.11	40	50.8	29.22	40	4.3%	17.50 [5.36, 29.64]
**Subtotal (95% CI)**			**40**			**40**	**4.3%**	**17.50 [5.36, 29.64]**
Heterogeneity: Not applicable								
Test for overall effect: Z = 2.82 (P = 0.005)								
**Total (95% CI)**			**90**			**90**	**100.0%**	**3.34 [0.81, 5.87]**
Heterogeneity: $Chi^2 = 5.46$, df = 1 (P = 0.02); $I^2 = 82\%$								
Test for overall effect: Z = 2.59 (P = 0.010)								
Test for subgroup differences: $Chi^2 = 5.46$, df = 1 (P = 0.02), $I^2 = 81.7\%$								

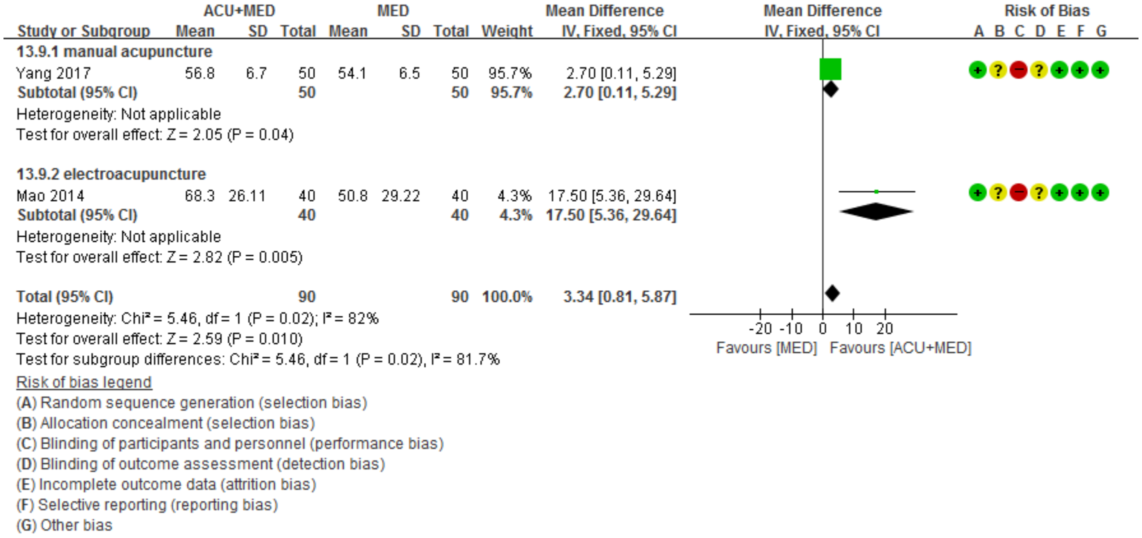

Risk of bias legend
(A) Random sequence generation (selection bias)
(B) Allocation concealment (selection bias)
(C) Blinding of participants and personnel (performance bias)
(D) Blinding of outcome assessment (detection bias)
(E) Incomplete outcome data (attrition bias)
(F) Selective reporting (reporting bias)
(G) Other bias

19) SF-36 social role functioning (subgroup analysis according to acupuncture type)

Study or Subgroup	ACU+MED Mean	ACU+MED SD	ACU+MED Total	MED Mean	MED SD	MED Total	Weight	Mean Difference IV, Fixed, 95% CI
**13.10.1 manual acupuncture**								
Yang 2017	60	7.1	50	58.5	6.9	50	84.9%	1.50 [-1.24, 4.24]
**Subtotal (95% CI)**			**50**			**50**	**84.9%**	**1.50 [-1.24, 4.24]**
Heterogeneity: Not applicable								
Test for overall effect: Z = 1.07 (P = 0.28)								
**13.10.2 electroacupuncture**								
Mao 2014	77.81	15.63	40	70.94	13.98	40	15.1%	6.87 [0.37, 13.37]
**Subtotal (95% CI)**			**40**			**40**	**15.1%**	**6.87 [0.37, 13.37]**
Heterogeneity: Not applicable								
Test for overall effect: Z = 2.07 (P = 0.04)								
**Total (95% CI)**			**90**			**90**	**100.0%**	**2.31 [-0.22, 4.84]**
Heterogeneity: Chi² = 2.23, df = 1 (P = 0.14); I² = 55%								
Test for overall effect: Z = 1.79 (P = 0.07)								
Test for subgroup differences: Chi² = 2.23, df = 1 (P = 0.14), I² = 55.1%								

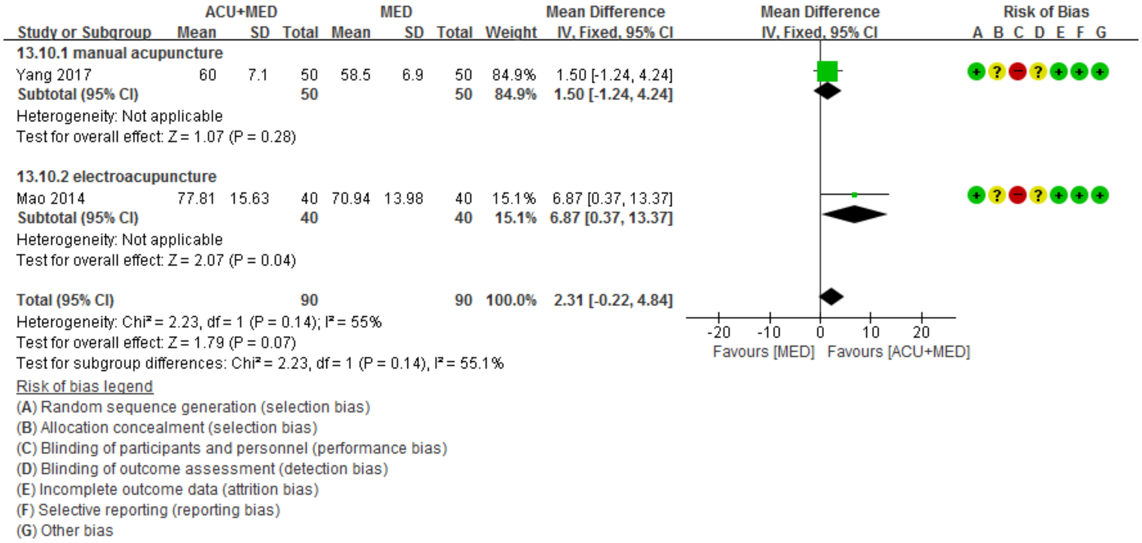

Risk of bias legend
(A) Random sequence generation (selection bias)
(B) Allocation concealment (selection bias)
(C) Blinding of participants and personnel (performance bias)
(D) Blinding of outcome assessment (detection bias)
(E) Incomplete outcome data (attrition bias)
(F) Selective reporting (reporting bias)
(G) Other bias

20) SF-36 mental health (subgroup analysis according to acupuncture type)

Study or Subgroup	ACU+MED Mean	ACU+MED SD	ACU+MED Total	MED Mean	MED SD	MED Total	Weight	Mean Difference IV, Fixed, 95% CI
**13.11.1 manual acupuncture**								
Yang 2017	63.6	7.4	50	55.8	6.6	50	82.5%	7.80 [5.05, 10.55]
**Subtotal (95% CI)**			**50**			**50**	**82.5%**	**7.80 [5.05, 10.55]**
Heterogeneity: Not applicable								
Test for overall effect: Z = 5.56 (P < 0.00001)								
**13.11.2 electroacupuncture**								
Mao 2014	62.6	13.93	40	51.6	13.28	40	17.5%	11.00 [5.04, 16.96]
**Subtotal (95% CI)**			**40**			**40**	**17.5%**	**11.00 [5.04, 16.96]**
Heterogeneity: Not applicable								
Test for overall effect: Z = 3.61 (P = 0.0003)								
**Total (95% CI)**			**90**			**90**	**100.0%**	**8.36 [5.86, 10.86]**
Heterogeneity: Chi² = 0.91, df = 1 (P = 0.34); I² = 0%								
Test for overall effect: Z = 6.56 (P < 0.00001)								
Test for subgroup differences: Chi² = 0.91, df = 1 (P = 0.34), I² = 0%								

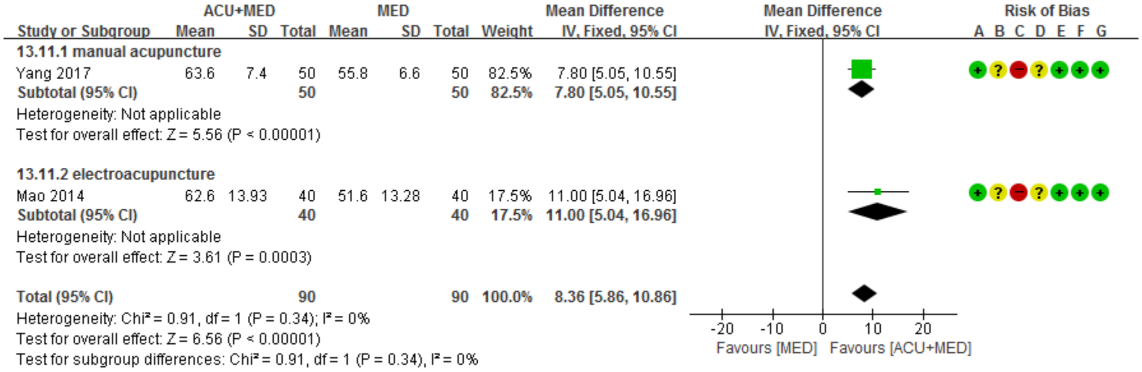

Risk of bias legend
(A) Random sequence generation (selection bias)
(B) Allocation concealment (selection bias)
(C) Blinding of participants and personnel (performance bias)
(D) Blinding of outcome assessment (detection bias)
(E) Incomplete outcome data (attrition bias)
(F) Selective reporting (reporting bias)
(G) Other bias

21) Recurrence rate (subgroup analysis according to WM type)

Study or Subgroup	ACU+MED Events	ACU+MED Total	MED Events	MED Total	Weight	Risk Ratio M-H, Fixed, 95% CI	A	B	C	D	E	F	G
**12.12.1 prokinetics**													
He 2012	7	130	15	130	44.5%	0.47 [0.20, 1.11]	?	?	-	?	+	+	+
Zhang 2014a	6	30	15	28	46.1%	0.37 [0.17, 0.83]	+	?	-	?	+	+	+
**Subtotal (95% CI)**		**160**		**158**	**90.6%**	**0.42 [0.23, 0.75]**							
Total events	13		30										
Heterogeneity: Chi² = 0.14, df = 1 (P = 0.71); I² = 0%													
Test for overall effect: Z = 2.90 (P = 0.004)													
**12.12.2 prokinetics&acid suppressants**													
Zhang 2014b	2	40	3	36	9.4%	0.60 [0.11, 3.39]	?	?	-	?	+	+	+
**Subtotal (95% CI)**		**40**		**36**	**9.4%**	**0.60 [0.11, 3.39]**							
Total events	2		3										
Heterogeneity: Not applicable													
Test for overall effect: Z = 0.58 (P = 0.56)													
**Total (95% CI)**		**200**		**194**	**100.0%**	**0.44 [0.25, 0.76]**							
Total events	15		33										
Heterogeneity: Chi² = 0.30, df = 2 (P = 0.86); I² = 0%													
Test for overall effect: Z = 2.92 (P = 0.003)													
Test for subgroup differences: Chi² = 0.15, df = 1 (P = 0.70), I² = 0%													

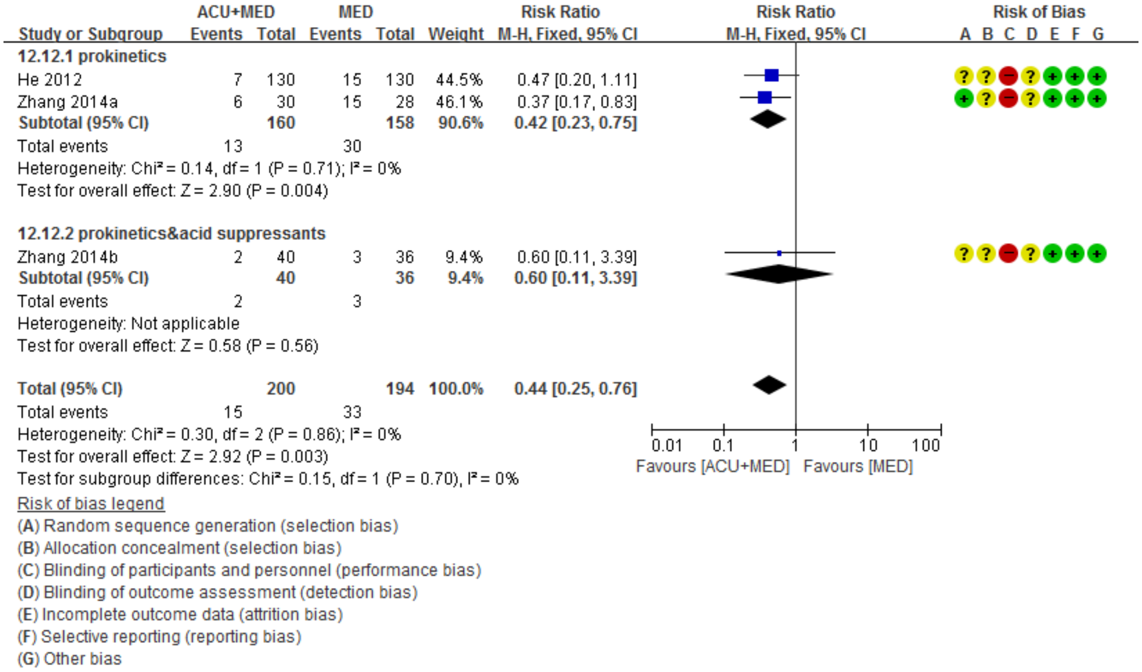

Risk of bias legend
(A) Random sequence generation (selection bias)
(B) Allocation concealment (selection bias)
(C) Blinding of participants and personnel (performance bias)
(D) Blinding of outcome assessment (detection bias)
(E) Incomplete outcome data (attrition bias)
(F) Selective reporting (reporting bias)
(G) Other bias

22) Recurrence rate (subgroup analysis according to acupuncture type)

Study or Subgroup	ACU+MED Events	ACU+MED Total	MED Events	MED Total	Weight	Risk Ratio M-H, Fixed, 95% CI	A	B	C	D	E	F	G
**13.12.1 manual acupuncture**													
He 2012	7	130	15	130	44.5%	0.47 [0.20, 1.11]	?	?	-	?	+	+	+
Zhang 2014a	6	30	15	28	46.1%	0.37 [0.17, 0.83]	+	?	-	?	+	+	+
Zhang 2014b	2	40	3	36	9.4%	0.60 [0.11, 3.39]	?	?	-	?	+	+	+
**Subtotal (95% CI)**		**200**		**194**	**100.0%**	**0.44 [0.25, 0.76]**							
Total events	15		33										
Heterogeneity: Chi² = 0.30, df = 2 (P = 0.86); I² = 0%													
Test for overall effect: Z = 2.92 (P = 0.003)													
**Total (95% CI)**		**200**		**194**	**100.0%**	**0.44 [0.25, 0.76]**							
Total events	15		33										
Heterogeneity: Chi² = 0.30, df = 2 (P = 0.86); I² = 0%													
Test for overall effect: Z = 2.92 (P = 0.003)													
Test for subgroup differences: Not applicable													

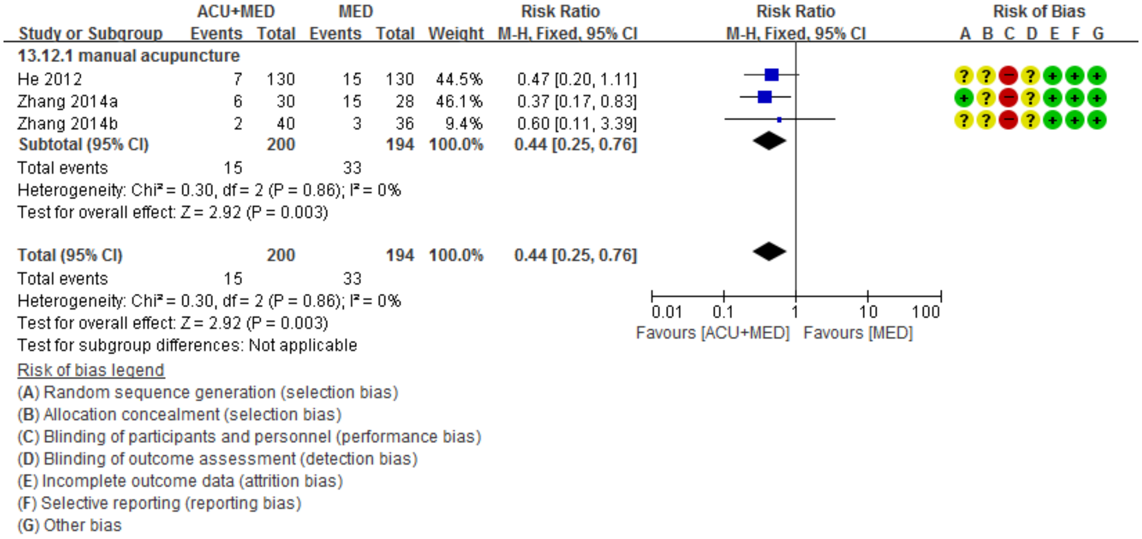

Risk of bias legend
(A) Random sequence generation (selection bias)
(B) Allocation concealment (selection bias)
(C) Blinding of participants and personnel (performance bias)
(D) Blinding of outcome assessment (detection bias)
(E) Incomplete outcome data (attrition bias)
(F) Selective reporting (reporting bias)
(G) Other bias

Supplement 5. Adverse events reported

Study or Subgroup	ACU+MED Events	ACU+MED Total	MED Events	MED Total	Weight	Risk Ratio M-H, Random, 95% CI
**11.1.1 P**						
Chen 2019	7	50	5	50	15.5%	1.40 [0.48, 4.12]
He 2012	14	130	16	130	18.6%	0.88 [0.45, 1.72]
Jiang 2017	2	45	4	45	11.4%	0.50 [0.10, 2.59]
Liu 2011	1	40	1	38	6.2%	0.95 [0.06, 14.65]
Mao 2014	2	40	2	40	9.8%	1.00 [0.15, 6.76]
Zhang 2013	0	36	0	36		Not estimable
Zhang 2014a	0	30	0	28		Not estimable
**Subtotal (95% CI)**		**371**		**367**	**61.4%**	**0.93 [0.56, 1.55]**
Total events	26		28			
Heterogeneity: Tau² = 0.00; Chi² = 1.14, df = 4 (P = 0.89); I² = 0%						
Test for overall effect: Z = 0.27 (P = 0.79)						
**11.1.2 acid**						
Zhang 2014b	2	40	3	36	10.8%	0.60 [0.11, 3.39]
**Subtotal (95% CI)**		**40**		**36**	**10.8%**	**0.60 [0.11, 3.39]**
Total events	2		3			
Heterogeneity: Not applicable						
Test for overall effect: Z = 0.58 (P = 0.56)						
**11.1.3 P+acid**						
Han 2020(part1)	0	39	1	40	5.0%	0.34 [0.01, 8.14]
**Subtotal (95% CI)**		**39**		**40**	**5.0%**	**0.34 [0.01, 8.14]**
Total events	0		1			
Heterogeneity: Not applicable						
Test for overall effect: Z = 0.66 (P = 0.51)						
**11.1.4 P+antidepressants**						
Yang 2016	0	50	0	50		Not estimable
**Subtotal (95% CI)**		**50**		**50**		**Not estimable**
Total events	0		0			
Heterogeneity: Not applicable						
Test for overall effect: Not applicable						
**11.1.5 P+acid+antidepressants**						
Han 2020(part2)	0	43	1	41	5.0%	0.32 [0.01, 7.59]
**Subtotal (95% CI)**		**43**		**41**	**5.0%**	**0.32 [0.01, 7.59]**
Total events	0		1			
Heterogeneity: Not applicable						
Test for overall effect: Z = 0.71 (P = 0.48)						
**11.1.6 gastrocaine**						
Chung 2019	41	66	6	66	17.8%	6.83 [3.11, 14.99]
**Subtotal (95% CI)**		**66**		**66**	**17.8%**	**6.83 [3.11, 14.99]**
Total events	41		6			
Heterogeneity: Not applicable						
Test for overall effect: Z = 4.79 (P < 0.00001)						
**Total (95% CI)**		**609**		**600**	**100.0%**	**1.13 [0.50, 2.54]**
Total events	69		39			
Heterogeneity: Tau² = 0.80; Chi² = 22.06, df = 8 (P = 0.005); I² = 64%						
Test for overall effect: Z = 0.29 (P = 0.77)						
Test for subgroup differences: Chi² = 20.45, df = 4 (P = 0.0004), I² = 80.4%						

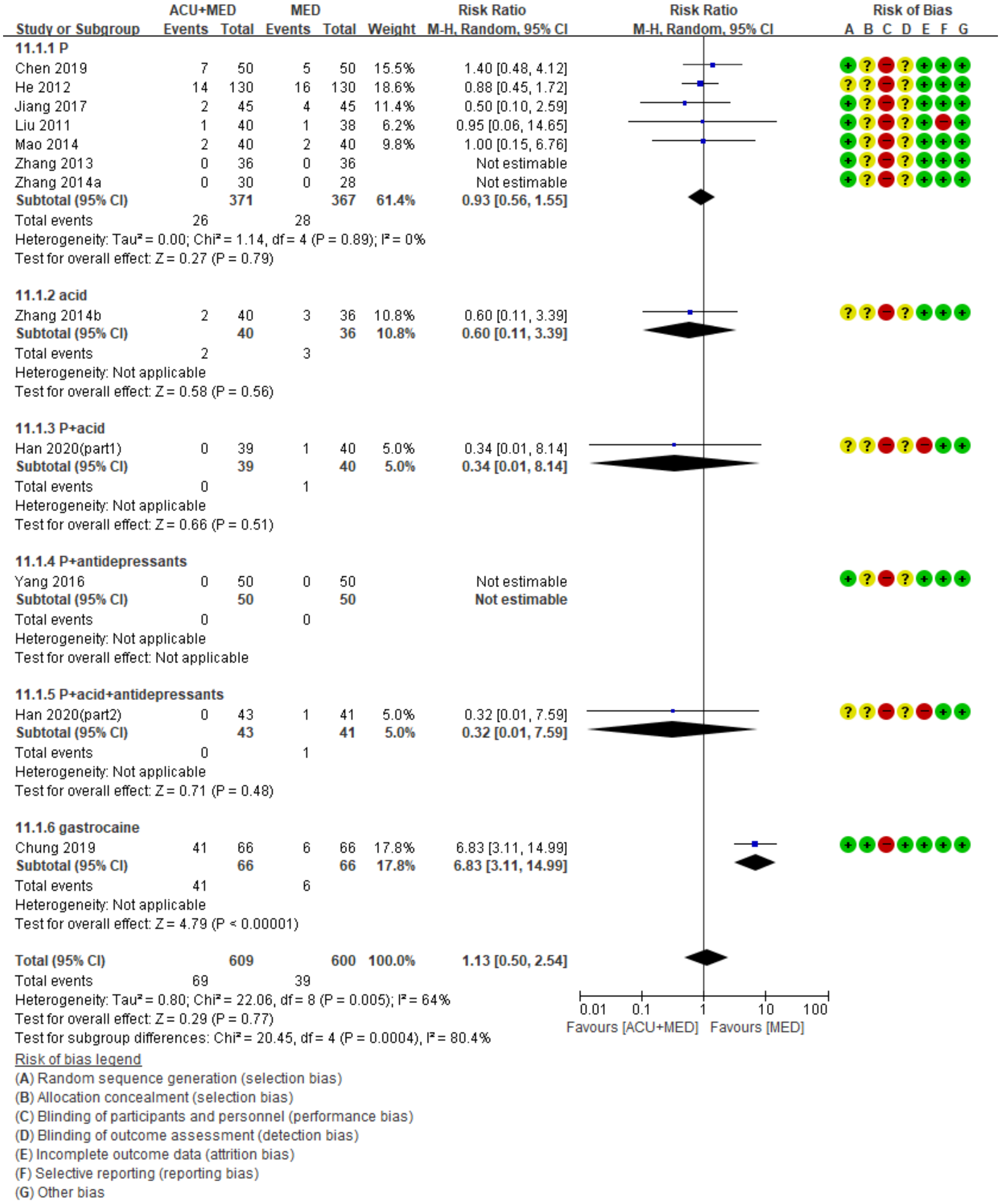

Risk of bias legend
(A) Random sequence generation (selection bias)
(B) Allocation concealment (selection bias)
(C) Blinding of participants and personnel (performance bias)
(D) Blinding of outcome assessment (detection bias)
(E) Incomplete outcome data (attrition bias)
(F) Selective reporting (reporting bias)
(G) Other bias

Abbreviations. Acid, acid suppressants; P, prokinetics.

Study or Subgroup	ACU+MED Events	ACU+MED Total	MED Events	MED Total	Weight	Risk Ratio M-H, Random, 95% CI
**11.7.1 MA**						
Chen 2019	7	50	5	50	15.5%	1.40 [0.48, 4.12]
He 2012	14	130	16	130	18.6%	0.88 [0.45, 1.72]
Jiang 2017	2	45	4	45	11.4%	0.50 [0.10, 2.59]
Liu 2011	1	40	1	38	6.2%	0.95 [0.06, 14.65]
Yang 2016	0	50	0	50		Not estimable
Zhang 2013	0	36	0	36		Not estimable
Zhang 2014a	0	30	0	28		Not estimable
Zhang 2014b	2	40	3	36	10.8%	0.60 [0.11, 3.39]
**Subtotal (95% CI)**		**421**		**413**	**62.5%**	**0.89 [0.54, 1.48]**
Total events	26		29			
Heterogeneity: Tau² = 0.00; Chi² = 1.35, df = 4 (P = 0.85); I² = 0%						
Test for overall effect: Z = 0.43 (P = 0.66)						
**11.7.2 EA**						
Chung 2019	41	66	6	66	17.8%	6.83 [3.11, 14.99]
Han 2020(part1)	0	39	1	40	5.0%	0.34 [0.01, 8.14]
Han 2020(part2)	0	43	1	41	5.0%	0.32 [0.01, 7.59]
Mao 2014	2	40	2	40	9.8%	1.00 [0.15, 6.76]
**Subtotal (95% CI)**		**188**		**187**	**37.5%**	**1.43 [0.26, 7.90]**
Total events	43		10			
Heterogeneity: Tau² = 1.83; Chi² = 8.57, df = 3 (P = 0.04); I² = 65%						
Test for overall effect: Z = 0.41 (P = 0.68)						
**Total (95% CI)**		**609**		**600**	**100.0%**	**1.13 [0.50, 2.54]**
Total events	69		39			
Heterogeneity: Tau² = 0.80; Chi² = 22.06, df = 8 (P = 0.005); I² = 64%						
Test for overall effect: Z = 0.29 (P = 0.77)						
Test for subgroup differences: Chi² = 0.27, df = 1 (P = 0.61), I² = 0%						

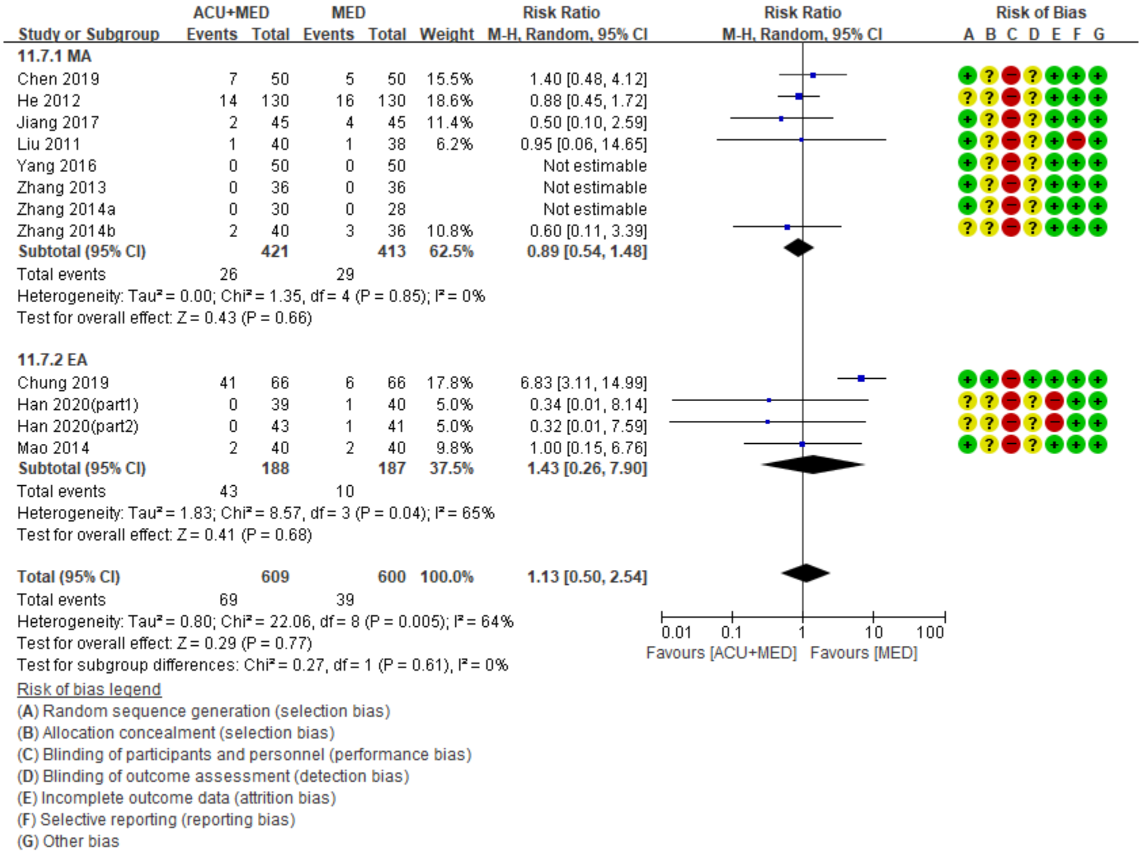

Risk of bias legend
(A) Random sequence generation (selection bias)
(B) Allocation concealment (selection bias)
(C) Blinding of participants and personnel (performance bias)
(D) Blinding of outcome assessment (detection bias)
(E) Incomplete outcome data (attrition bias)
(F) Selective reporting (reporting bias)
(G) Other bias

Abbreviations. EA, electro-acupuncture; MA, manual acupuncture.

Study ID	Adverse events
Chen 2011	NR
Chen 2012	NR
Chen 2017	NR
Chen 2019	(A) anorexia (2 cases), lethargy (2 cases), sweating (2 cases), dizziness (1 case)<br>(B) anorexia (2 cases), lethargy (1 case), sweating (1 case), dizziness (1 case)
Chung 2019	(A) EA-related: pain at acupoints (15 cases), bruising at acupoints (10 cases), numbness and tingling at acupoints (7 cases), worsening epigastric pain and fullness (4 cases), swelling at acupoints (2 cases), dizziness (1 case), muscle spasm (1 case), insomnia (1 case); Gastrocaine-related: epigastric discomfort (1 case), facial acne (1 case)<br>(B) dry mouth (2 cases), epigastric burning and pain (1 case), loss of appetite (1 case), dizziness (1 case), bitter taste in mouth and mild diarrhea (1 case)
Fan 2015	NR
Gao 2016	NR
Han 2020a	(B) mild diarrhea (1 case)
Han 2020b	(B) mild diarrhea (1 case)
He 2012	(A) mild anorexia (5 cases), increased bowel frequency (4 cases), rugitus (5 cases)<br>(B) anorexia (6 cases), increased bowel frequency (5 cases), rugitus (5 cases)
Jiang 2017	(A) diarrhea (2 cases)<br>(B) diarrhea (4 cases)
Liu 2011	(A) diarrhea (1 case)<br>(B) diarrhea (2 cases)
Mao 2014	(A) ecchymosis (2 cases)<br>(B) dry mouth and fatigue (1 case), mild diarrhea (1 case)
Mei 2018	NR
Wang 2019	NR
Yan 2016	NR
Yang 2016	None
Yang 2017	NR
Yu 2008	NR
Zhang 2010	NR
Zhang 2013	None
Zhang 2014a	None
Zhang 2014b	(A) headache (1 case), dry mouth (1 case)<br>(B) diarrhea (1 case), headache (2 cases)

Abbreviations. EA, electro-acupuncture; NR, not recorded.

Note. (A) treatment group, (B) control group.
